# M6A-mediated molecular patterns and tumor microenvironment infiltration characterization in nasopharyngeal carcinoma

**DOI:** 10.1080/15384047.2024.2333590

**Published:** 2024-03-26

**Authors:** Yong Wang, Lisha Peng, Feng Wang

**Affiliations:** Department of Radiotherapy, The First Affiliated Hospital of Kunming Medical University, Kunming, Yunnan, China

**Keywords:** M6A modification, tumor microenvironment, molecular pattern, prognosis, nasopharyngeal carcinoma

## Abstract

N6-methyladenosine (m6A) is the most predominant RNA epigenetic regulation in eukaryotic cells. Numerous evidence revealed that m6A modification exerts a crucial role in the regulation of tumor microenvironment (TME) cell infiltration in several tumors. Nevertheless, the potential role and mechanism of m6A modification in nasopharyngeal carcinoma (NPC) remains unknown. mRNA expression data and clinical information from GSE102349, and GSE53819 datasets obtained from Gene Expression Omnibus (GEO) was used for differential gene expression and subsequent analysis. Consensus clustering was used to identify m6A-related molecular patterns of 88 NPC samples based on prognostic m6A regulators using Univariate Cox analysis. The TME cell-infiltrating characteristics of each m6A-related subclass were explored using single-sample gene set enrichment (ssGSEA) algorithm and CIBERSORT algotithm. DEGs between two m6A-related subclasses were screened using edgeR package. The prognostic signature and predicated nomogram were constructed based on the m6A-related DEGs. The cell infiltration and expression of prognostic signature in NPC was determined using immunohistochemistry (IHC) analysis. Chi-square test was used to analysis the significance of difference of the categorical variables. And survival analysis was performed using Kaplan–Meier plots and log-rank tests. The NPC samples were divided into two m6A-related subclasses. The TME cell-infiltrating characteristics analyses indicated that cluster 1 is characterized by immune-related and metabolism pathways activation, better response to anit-PD1 and anti-CTLA4 treatment and chemotherapy. And cluster 2 is characterized by stromal activation, low expression of HLA family and immune checkpoints, and a worse response to anti-PD1 and anti-CTLA4 treatment and chemotherapy. Furthermore, we identified 1558 DEGs between two m6A-related subclasses and constructed prognostic signatures to predicate the progression-free survival (PFS) for NPC patients. Compared to non-tumor samples, REEP2, TMSB15A, DSEL, and ID4 were upregulated in NPC samples. High expression of REEP2 and TMSB15A showed poor survival in NPC patients. The interaction between REEP2, TMSB15A, DSEL, ID4, and m6A regulators was detected. Our finding indicated that m6A modification plays an important role in the regulation of TME heterogeneity and complexity.

## Introduction

Nasopharyngeal carcinoma (NPC) is a subtype of head and neck tumor, which is an epithelial carcinoma that originates from nasopharyngeal mucosal lining and is different from other head and neck tumors.^1^ Based on the morphologic characteristics, NPC is divided into epithelial carcinoma (EC), sarcomatoid carcinoma (SC), mixed sarcomatoid-epithelial carcinoma (MSEC), and squamous cell carcinoma (SCC).^2^ NPC is a rare tumor comparison with other tumors worldwide, which is 129,079 new cases and 73,987 deaths in 2018.^3^ However, the distribution of NPC unevenly around the world, which highly occurs in east and southeast Asia, with 113,659 (85.5%) new cases in Asia, and 62,44 (46.8%) in China in 2020.^3–5^ The geographical distribution of NPC is also extremely unbalanced in China, higher in South China than in other regions.^6^ Even with advanced regimens, such as liquid biopsies, minimally invasive surgery, radiotherapy, chemotherapy, and immunotherapy widely used for NPC treatment,^7–9^ approximately 10–20% of NPC patients suffer recurrence after primary treatment.^10,11^ Also, the asymptomatic nature of NPC hinders the early diagnose of NPC. NPC is a heterogeneous tumor with complexity contributed to the risk factors including genomic variations, Epstein-Barr virus (EBV) infection, smoking, diet, gender, environmental, and other clinical characteristics.^12,13^ Therefore, finding the novel marker is of important in the early diagnose and treatment of NPC.

Tumor microenvironment (TME) consists of cancer cells, variation immune cells, stromal cells, bone marrow-derived cells, and they secreted cytokine, chemokines, and growth factors,^14^ and is characterized by hypoxia, high oxidation, acidity, malnutrition, and inflammation because of the heterogenous of cells in TME.^15,16^ TME associates to hall makers of cancer,^17^ radio-/chemoresistance,^18,19^ immune evasion and immunotherapeutic response,^20^ tumor progression and recurrence,^21^ and prognosis.^22^ When it refers to NPC, NPC has the most severe stromal infiltration compared to other solid tumors, and the pathological status might alter cellular composition in the NPC microenvironment.^23^ Liu et al.^24^ found that in EBV-induced NPC, the cell infiltration of CD8+ T cells cells and NK cells were limited. In contrast, regulatory T cells (Treg), M2 macrophage, and B cells can promote tumor cell proliferation by inhibiting the activity of CD8+ T cells and promote metastasis, while tumor endothelial cells (TEC) and cancer-associated fibroblasts (CAF) were increased. TME exert a important role in tumor growth and development and its composition needs to be investigated in more depth.

N6-methyladenosine (m6A) is an RNA modification in eukaryotic cells, which emerges as a widespread regulatory mechanism, such as regulating RNA transcription, processing, splicing, degradation, translation, export, and stability in diverse physiological processes.^25,26^ M6A modified on RNA is dynamically regulated by m6A regulators that contain adenosine methyltransferases (RBM15, ZC3H13, METTL3, METTL14, WTAP, and KIAA1429), m6A-binding proteins (YTHDF1/2/3, YTHDC1/2, HNRNPA2B1, LRPPRC, FMR1), and demethylases (FTO and ALKBH5), as known as writers, readers, and erasers.^26,27^ Increasing shreds of evidence strongly indicate that m6A modification acts as a dominant role in regulating tumor progression, chemoresistance, immunotherapeutic response, and prognosis.^28–31^ M6A modification has been proved that significantly associated with tumorigenesis, metastasis, and progression.^32,33^ M6A modification plays a nonnegligible role in the diversity and complexity of TME.^34,35^ Interaction between m6A modification and TME mediates the biological processes of cancer cells, immune cells, and stromal cells to influence tumor initiation, progression, and therapy responses.^36–38^ Understanding the interaction between m6A modification and TME is of great significance to developing effective therapeutic strategies and estimating the prognosis. Although some researches have revealed the role of m6A modification in tumorigenesis, progression, and therapy response,^39–41^ the systematic analysis of the interaction between m6A modification and TME in NPC remains unclear.

In the present study, we systematically collected mRNA and corresponding clinical information of NPC patients to identify the m6A-related molecular patterns in NPC explored the TME cell infiltrating characteristics, and predicated the therapeutic response for each subclass. Moreover, we also constructed an m6A-related prognostic risk model and identify the prognostic signature, then developed a predicated nomogram to predicate progression-free survival (PFS) for NPC patients. Our finding might provide the potential biomarkers for NPC diagnosis and treatment, and supply the evidence for managing the therapeutic strategy.

## Methods

### Data downloading and processing

mRNA expression data of NPC patients of two datasets, GSE102349, and GSE53819 datasets, were obtained from Gene Expression Omnibus (GEO, https://www.ncbi.nlm.nih.gov/geo/). The mRNA expression data of the GSE102349 dataset (Submission date: Aug 08, 2017, Last update date: May 15, 2019, data retrieval: Jan 08, 2022)^42^ was generated using Illumina HiSeq 2000, this dataset includes mRNA expression data and clinical information from 113 NPC patients, and 88 cases with progression-free survival (PFS) involved in this study. And the mRNA expression data of the GSE53819 dataset (Submission date: Jan 04, 2014, Last update date: Aug 01, 2019, data retrieval: Jan 08, 2022)^43^ was generated using Agilent -014,850 Whole Human Genome Microarray 4 × 44K G4112F, and this dataset contained 18 NPC primary tumor tissues and 18 non-cancerous nasopharyngeal tissues.

### Construction of prognosis-related signature based on 21 m6A regulators

Univariate Cox analysis was performed with survival R package to identify the m6A regulators with prognostic significance in NPC, the significant m6A regulators were identified using univariate Cox analysis of PFS with *P*-value <.05. Here, 21 m6A regulators, including 8 writers (METTL3, METTL14, RBM15, RBM15B, WTAP, KIAA1429, CBLL1, ZC3H13), 2 erasers (ALKBH5, FTO), and 11 readers (YTHDC1, YTHDC2, YTHDF1, YTHDF2, YTHDF3, IGF2BP1, HNRNPA2B1, HNRNPC, FMR1, LRPPRC, ELAVL1), were involved in this conducted.

### Identification and validation of the molecular subclusters

The unsupervised class was a powerful technique in cancer research, and the consensus clustering methods were used for estimating the number of unsupervised classes in a dataset.^44^ The consensus clustering was performed using the consensusClusterPlus package in R to conduct the classification in the GSE102349 dataset (88 NPC patients) according to their expression profiles of prognosis-related m6A regulators. The *k* value was determined with consistent cumulative distribution function (CDF) reached an approximate maximum, that represented the optimal number of clusters. T-Distributed Stochastic Neighbor Embedding (t-SNE) is a non-linear dimensionality reduction method with minimum structural information loss and is widely used for bioinformatics.^45^ In this study, t-SNE was performed to validate the subtype assignments using mRNA expression data of GSE102349 and GSE53819 datasets. Moreover, a Submap matrix was conducted to detect the similarity of molecular classes from GSE102349 and GSE53819 datasets.^46^ In addition, differential expression of the 10 m6A regulators between subclusters was detected and visualized using pheatmap package in R.

### Gene set variation analysis (GSVA) for function enrichment

GSVA is a Gene set enrichment (GSE) method that estimates variation pathway and biological processes activity over a sample population in a non-parametric and unsupervised method.^47^ Here, we conducted the GSVA using GSVA package in R to investigate the various biological function among distinct m6A subclasses. The Kyoto Encyclopedia of Genes and Genomes (KEGG) pathway gene set (c2.cp.kegg.v7.4.symbols.gmt) and 50 human cancer gene sets (h.all.v7.4.symbols.gmt) were downloaded from Molecular Signatures Database (MSigDB, data retrieval: Jan 08, 2022, https://www.gsea-msigdb.org/gsea/msigdb/index.jsp), Biological process gene set (c5.go.bp.v7.4.symbols.gmt) was downloaded from Gene Ontology (GO) database (data retrieval: Jan 08, 2022, http://geneontology.org/), and pathway gene set (c2.cp.reactome.v7.4.symbols.gmt) was obtained from Reactome pathway database (data retrieval: Jan 08, 2022, https://reactome.org/). Besides, we also obtained the gene sets that related to CD8 T effector, DNA damage repair, Nucleotide excision repair from MSigDB, and a previous study.^48^ Above gene sets were performed GSVA to identify the enriched biological processes and pathways. Finally, the various biological processes and pathways between distinct subclusters were determined by Wilcox rank-sum or Kruskal–Wallis tests. Adjusted *P*-value <.05 was considered statistically significant.

### Estimate tumor immune cell infiltration

We used the ESTIMATE algorithm to estimate the composition of the tumor microenvironment (TME).^49^ The ratio of the immune cells and stromal cells in the TME was quantized with the immune score and stromal score. The differences between distinct subclasses were detected by Wilcox rank-sum or Kruskal–Wallis tests. In addition, we used the single-sample gene set enrichment (ssGSEA) algorithm^50^ to estimate TME immune cell infiltration according to the marker genes of 24 immune cell types.^51^ And the relative abundance of each cell infiltration was quantized by conducting ssGSEA using GSVA package in R. the variation between m6A-related subclusters were examined by Wilcox rank-sum or Kruskal–Wallis tests. Also, gene expression data from GSE102349 was used to assess the relative abundance of 22 immune infiltration in two subclasses. Also, the the difference in immune cell infiltration was tested using the Wilcoxon test, and the difference was considered significant when *p* < .05.

### Evaluation of the response for immunotherapy of each subclass

Human leukocyte antigen (HLA) genes and immune checkpoints exert a key role in response for immunotherapy. in this study, we detected the expression of HLA genes (HLA-A/B/C/E/F/G/DRB1/DQB1/DQA1/DPB1/DRA/DRB5/DPA1/DMA/DMB/DQA2/DOA/DQB2/DOB) and immune checkpoints (IDO1, PD-L1 (CD274), PD-L2 (PDCD1LG2), TIM-3 (HAVCR2), PD-1 (PDCD1), CTLA-4, CD80, CD86) between m6A-related subclasses. Furthermore, tumor immune dysfunction and exclusion (TIDE) and the SubMap algorithm were used to predict the immune response to checkpoint inhibitors PD-1 and CTLA4 in distinct m6A-related subclasses. TIDE is a computational method to predicate the immune checkpoint blocked (ICB) response based on two primary mechanisms of tumor immune evasion, including the induction of T cell dysfunction in tumors with high cytotoxic T lymphocytes (CTL) levels and the prevention of T cell infiltration in tumor with low CTL level.^52^ The TIDE score reflects the efficiency of the immunotherapeutic outcome, high TIDE score indicates a poor treatment response rate.^53^ Moreover, the Submap mapping was used to compare the response for anti-PD-1 and anti-CTLA4 treatment between m6A-related subclusters. *P*-value <.05 and Bonferroni corrected *P*-value <.05 considered statistically significant.

### Evaluation of the response for chemotherapy of each subclass

We also investigated the chemotherapy response rate using pRRophetic package in R. According to the Genomics of Drug Sensitivity in Cancer (GDSC) database (https://www.cancerrxgene.org/),^53^ pRRophetic algorithm was conducted to identify the antitumor drugs from 138 common antitumor drugs using Ridge’s regression with the half-maximum inhibitory concentration (IC50) of each patient. The low IC50 value indicated a high response for chemotherapy.

### Characterization of m6A-based subclasses in NPC

The differentially expressed genes (DEGs) among NPC subclasses were identified using edgeR package with parameters of |log2 (fold change, FC) |>1 and FDR < 0.05. ggplot2 and pheatmap R packages were used to visualize the DEGs among NPC subclasses.

### Biofunction enrichment of DEGs of m6A-based subclass

GO and KEGG pathway enrichment was conducted using clusterProfiler package in R with *P*-value <.05 or *q*-value <0.05. GO annotation included biological process (BP), molecular function (MF), and cellular components (CC) terms.

### Construction of the m6A-related gene signature and risk model

88 samples in the GSE102394 dataset were divided into a training set and test set at a 7:3 ratio. The expression data of DEGs among m6A-related subclasses and their corresponding clinical information were used for univariate Cox and Kaplan–Meier analyses with *P*-value <.05. Then, the m6A-related genes related to prognosis were identified using LASSO regression analysis using glmet package in R. The penalty parameter (λ) of the model was confirmed by ten-fold cross-validation. To estimate the prognostic value of the above prognostic signature, the risk score of the training set, test set, and whole set was calculated according to the formula: $\mathrm{risk}\;\mathrm{score}=\;\overset{n}{\underset{i=1}{\sum }}\mathrm{coef}\left(\mathrm{genei}\right)*\mathrm{expr}\left(\mathrm{genei}\right)$, coef represented the risk coefficient of each gene, expr represented an expression of each gene. All samples of the training set, test set, and whole set were divided into the high-risk score and low-risk score groups according to the median risk score. Kaplan–Meier analysis with log-rank test was used to compare the PFS between high-risk score and low-risk score groups. And the time-dependent receiver operating characteristic (ROC) curve was constructed using the survivalROC R package to estimate the accuracy of the prognostic model. The area under curve (AUC) have reflected the accuracy of the ROC result.

### Development of a nomogram for prognosis predicating

A nomogram predicted model was constructed based on the risk factors using rms package in R. and the prediction accuracy of the nomogram was estimated using the calibration curve. Moreover, we also investigated the correlation between prognostic factors and m6A regulators using Pearson correlation analysis. And *P*-value < 5 and correlation coefficient > 0.3 considered significant correlation.

### Gene Set Enrichment Analysis (GSEA)

Single gene GSEA was conducted in accordance with KEGG (c2.cp.kegg.v2022.1.Hs.entrez.gmt) gene sets in GSEA database (http://www.gsea-msigdb.org/gsea/msigdb, data retrieval: Nov 10, 2023) using clusterProfilerj and org.Hs.eg.db package in R script. The sample was classified as high- and low-gene expressed group referred to the expression of REEP2, TMSB15A, DSEL, and ID4, and spearman correlation was calculated between the expression of m6A-related prognostic signature and other genes in the dataset. The pathway was selected when padjust < 0.05 and |NES| > 1, and TOP 10 pathways for each gene was selected according to padjust.

### Transcription factor (TF) prediction

REEP2, TMSB15A, DSEL, and ID4 was entered into NetworkAnalyst Database (https://www.networkanalyst.ca/NetworkAnalyst/, data retrieval: Nov 10, 2023), and JASPER database was for TFs prediction was selected. Cytoscape (version 3.9.1) was used to plot the interaction network of hub genes and TFs.

### Clinical samples

A total of 255 primary NPC samples and adjacent normal nasopharyngeal samples were collected from the First Affiliated Hospital of Kunming Medical University. None of the patients had received chemotherapy or radiotherapy before surgery. All patients involved in this study were known the purposes of this study and wrote the informed consent. Our study was approved by the Ethics Committee of the First Affiliated Hospital of Kunming Medical University. The clinical characteristics of these patients were summarized in Table 1. All samples were quickly frozen in liquid nitrogen and used for subsequent experiments.

**Table 1.** Clinical characteristics of the NPC patients.

Characteristics	Number (*n* = 255)
Gender	
Male	169
Female	86
Age	49.4 ± 11.46
<60 years	127
≥60 years	128
Tumor size	2.86 ± 1.90
<4	189
≥4	66
T stage	
T1-T2	126
T3-T4	129
N stage	
N0-N1	90
N2-N3	165
M stage	
M0	240
M1	15
Smoking	
Yes	122
No	133

### Immunohistochemistry (IHC)

Paraffin sections were deparaffinized and hydrated with xylene and gradient concentration of ethanol (100%, 95%, 85%, and 70%). Then, antigen retrieval was achieved by boiling the sections in 10 mM sodium citrate for 10 min in a microwave oven. The sections were then washed with PBS three times and treated with 3% H_2_O_2_-methanol for 15 min. Immunostaining was performed by incubation with antibody against DSEL (1:150, NBP2–56370, Novus Biologicals), ID4 (1:100, LS‑B9923, Lsbio), REEP2 (1:100, bs -15,198 R, bioss), TMSB15A (1:100, sc -271,649, Santa Cruz Biotechnology), CD20 (1:150, ab78237, abcam), CD27 (1:250, ab131254, abcam), CD68(1:250, ab131254, abcam) CD4 (1:100, ab133616, abcam), CD45RO (1:100, ab23, abcam), CD8 (1:100, ab23, abcam) for 2 h. The sections were subsequently incubated with secondary antibody labeled with horseradish peroxidase for 30 min at room temperature. Cells with brown cytoplasm were counted as positive cells.

### Statistical analysis

In this study, all statistical analyses and visualized were performed using R software version 3.4.4 and GraphPad Prism 8 software. The continuous variables were shown as mean ± standard deviation (SD), Chi-square test was used to analysis the significance of difference of the categorical variables. And survival analysis was performed using Kaplan–Meier plots and log-rank tests. p-value <.05 was considered statistical significance.

## Results

### Construction of two m6A-related subclasses in NPC

**Figure 1.**
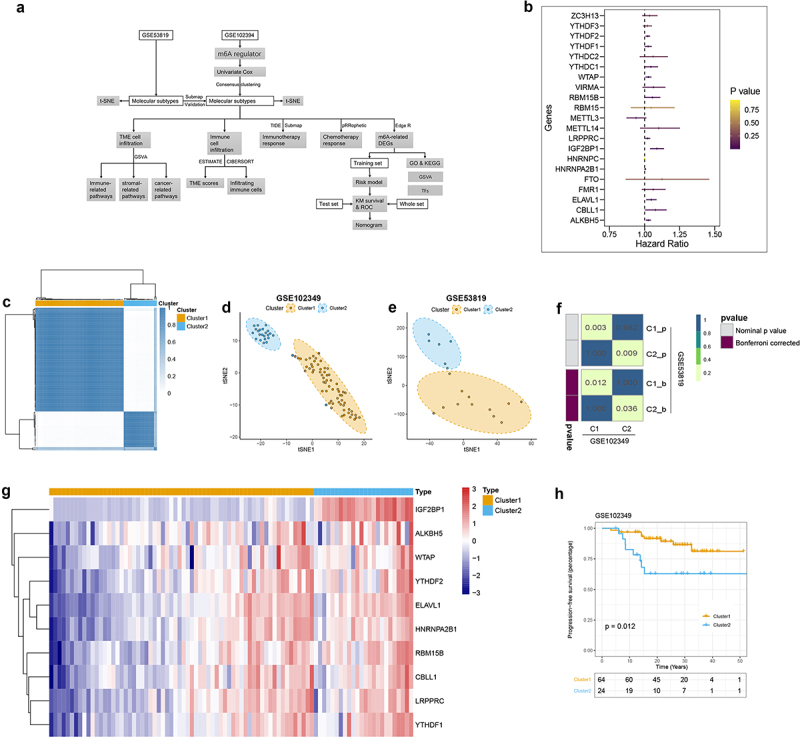
Construction of two m6A-related subclasses in NPC A. The workflow of this study. B. Forest plot showing the results of the Univariate Cox regression analysis between m6A regulators and PFS. C. Consensus matrices of the GSE102349 dataset for k = 2. D-E. T-SNE analyses show the stratification into two m6A-related subclasses of GSE102349 and GSE53819 datasets. F. Submap matrix analysis validated the similarity of molecular classes from GSE102349 and GSE102349 datasets.G. The expression of 10 prognostic-related m6A regulators (IGF2B1, ALKBH5, YTHDF2, ELAVL1, WTAP, LRPPRC, HNRNPA2B1, CBLL1, RBM15B, YTHDF1) between two m6A-related subclasses. H. Kaplan-meier survival plot showing the PFS between two m6A-related subclasses.

The systematic analyses of this study have been illustrated in Figure 1a. We obtained the mRNA expression files and corresponding clinical information of 88 NPC patients from the GEO database. Twenty-one m6A regulators were identified in this study from the above dataset. According to the results of Univariate Cox, we found 10 regulators (IGF2B1, ALKBH5, YTHDF2, ELAVL1, WTAP, LRPPRC, HNRNPA2B1, CBLL1, RBM15B, YTHDF1) significantly associated with PFS (Figure 1b, Table 2). Therefore, we clustered NPC patients into different subclasses according to the expression of the 10 m6A regulators who with prognostic values. As shown in Figure 1c and Figure S1A, all patients in the GSE102349 dataset were divided into two distinct subclasses based on the molecular pattern of m6A regulators by consensus clustering, including cluster 1 (64 patients) and cluster 2 (24 patients). The clinical characteristics of 88 patients from the GSE102349 dataset were shown in Table 3. T-SNE was performed to validate the above clustering by reducing the dimension of the feature, resulting in that the distinct assignment similar to consensus clustering (Figure 1d). Besides, we performed the clustering analysis in another independent dataset (GSE53819), the results indicated that there are also two distinct subclasses of NPC in the GSE53819 dataset (Figure S1B). And the t-SNE results are concordant with consensus clustering results (Figure 1e). Moreover, a SubMap analysis was conducted to validate the correlation between the subclasses from different datasets, the results indicated that C1 and C2 subclasses in the GSE102349 dataset were significantly correlated with the subclasses in the GSE53819 dataset (Figure 1f, Figure S1C). These results suggested that there were two distinct subclusters of NPC with the different m6A regulator patterns. We also found 10 m6A regulators upregulated in cluster 2 compared with cluster 1 (Figure 1g, Table S1). Kaplan-Meier PFS indicated the clinical relevance of m6A regulators of two m6A-based subclasses in NPC, suggesting that NPC patients belong to cluster 2 with poor prognosis than the patients in cluster 1 (Figure 1h).

**Table 2.** Identification of 21 m6A regulators with prognostic significance in the GSE102349 dataset based on Univariate Cox analysis.

Gene	HR	HR.95 L	HR.95 H	Cox p value
IGF2BP1	1.087891	1.039377	1.13867	0.000295
ALKBH5	1.026407	1.007793	1.045364	0.00525
YTHDF2	1.022635	1.005949	1.039599	0.007664
ELAVL1	1.04798	1.011201	1.086097	0.010139
WTAP	1.026768	1.005417	1.048573	0.013747
LRPPRC	1.022022	1.003458	1.040929	0.019855
HNRNPA2B1	1.004968	1.000487	1.009469	0.029733
CBLL1	1.077413	1.002618	1.157788	0.042236
RBM15B	1.055839	1.001878	1.112706	0.042349
YTHDF1	1.025377	1.000221	1.051167	0.047997
YTHDC1	1.045123	0.996353	1.09628	0.070281
METTL3	0.939385	0.870961	1.013185	0.105127
VIRMA	1.063797	0.985365	1.148472	0.113496
FMR1	1.06168	0.980282	1.149837	0.141388
METTL14	1.100512	0.967543	1.251756	0.144909
ZC3H13	1.035873	0.982245	1.092428	0.193792
YTHDC2	1.060862	0.965121	1.1661	0.22084
YTHDF3	1.019999	0.98803	1.053002	0.222928
FTO	1.124347	0.865234	1.461055	0.380537
RBM15	1.046799	0.901869	1.215019	0.547486
HNRNPC	1.000255	0.993944	1.006607	0.936992

**Table 3.** Clinical characteristics of NPC patients from GSE102349 dataset in distinct subclasses.

Clinical Stage	Cluster 1 (*n* = 64)	Cluster 2 (*n* = 24)
I	5 (7.8%)	0 (0%)
II	1 (1.6%)	1 (4.2%)
III	27 (42.2%)	12 (50.0%)
IV	15 (23.4%)	7 (29.2%)
Unknown	16 (25.0%)	4 (16.7%)
Morphology		
differentiated	7 (10.9%)	3 (12.5%)
mixed (round & spindle)	21 (32.8%)	11 (45.8%)
N/A	9 (14.1%)	1 (4.2%)
undifferentiated, round	21 (32.8%)	7 (29.2%)
undifferentiated, spindle	6 (9.4%)	2 (8.3%)
Intratumoral Tils %		
<30	24 (37.5%)	15 (62.5%)
≥30	33 (51.6%)	8 (33.3%)
Not evaluated	7 (10.9%)	1 (4.2%)
Stromal Tils %		
<50	23 (35.9%)	12 (50.0%)
≥50 & <70	7 (10.9%)	4 (16.7%)
≥70	27 (42.2%)	7 (29.2%)
Not evaluated	7 (10.9%)	1 (4.2%)

### Characteristics analysis of TME cell infiltration in two m6A-related subclasses

**Figure 2.**
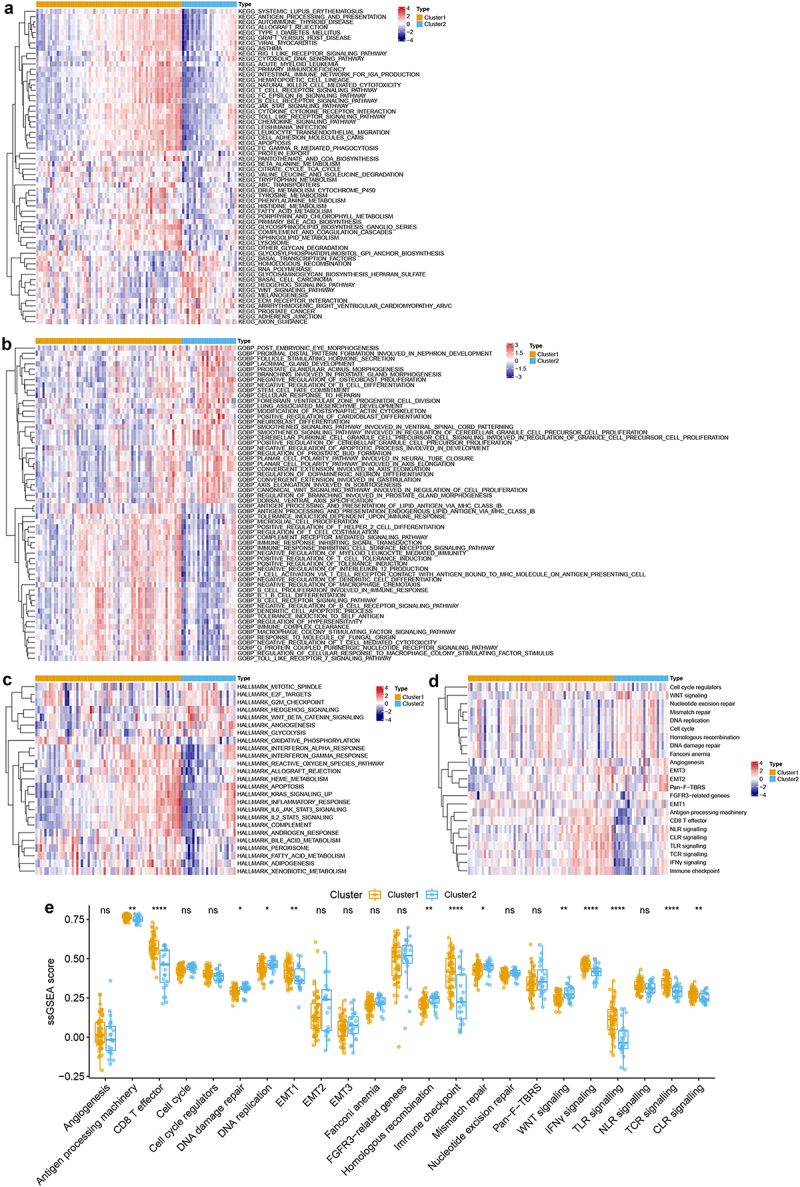
Characteristics analysis of tme cell infiltration in two m6a-related subclasses a. heatmap showing gsva enrichment analysis of the various pathways between two m6a-related subclasses. b. heatmap showing gsva enrichment analysis of the biological processes between two m6a-related subclasses.C. heatmap showing gsva enrichment analysis of 50 hallmark cancer-related pathways between two m6a-related subclasses.D. heatmap showing gsva enrichment analysis of oncogenic and TME cell-infiltrating related pathways between two m6a-related subclasses.E. boxplot showing the distinction of oncogenic and TME cell-infiltrating related pathways between two m6a-related subclasses.

GSVA was performed to investigate variation pathways among distinct m6A subclasses. We firstly explored the pathways enriched in each subclass, as shown in Figure 2a and Table S2, there were distinct pathways enriched in two m6A-related subclasses. The special genes of cluster 1 were primarily enriched in 46 pathways, most of those pathways were immune-related pathways and some metabolism pathways. The significant immune-related pathways which enriched in immune-related pathways included the B cell receptor signaling pathway, T cell receptor signaling pathway, chemokine signaling pathway, natural killer cell-mediated cytotoxicity, and Toll-like receptor signaling pathway, antigen processing, and presentation. And the metabolism pathways included fatty acid metabolism, beta-alanine metabolism, tyrosine metabolism cytochrome P450, porphyrin, and chlorophyll metabolism. However, the special genes of cluster 2 enriched in 14 pathways, including the stromal and tumorigenic pathways, such as ECM receptor interaction, Wnt signaling pathway, adheres junction, Hedgehog signaling pathway. These results indicated that stromal activation in cluster 2 might inhibit the antitumor effects of immune cells and increase immune escape. Whereas immune-related pathways activation in cluster 1 might imply the sensitivity to antitumor therapy. Moreover, we also identify 301 pathways enriched in cluster 1 and 211 pathways enriched in cluster 2 based on Reactome database (Table S3), that accordance to above results which were the special genes of cluster 1 primarily enriched in immune-related pathways and the special genes of cluster 2 mostly enriched in stromal-related pathways.

Additionally, GO enrichment analysis was performed using GVSA, resulting in the special genes of cluster 1 enriched in immune-related biological processes, such as antigen processing and presentation endogenous lipid antigen via MHC class IB, B cell differentiation, T cell activation, immune response, positive regulation of macrophage chemotaxis, positive regulation of T help 2 cell differentiation, etc. Inversely, the special genes of cluster 2 enriched in negative regulation of B cell differentiation, stem cell fate commitment, etc. (Figure 2b, Table S4).

We further explored the correlation between m6A-related molecular patterns and 50 Hallmark cancer-related pathways, and we found that the special genes of cluster 1 mostly enriched in 18 pathways, such as interferon-alpha/gamma (INF-α/γ) response, IL-6/JAK/STAT3 signaling pathway, inflammatory response, IL2/STAT5 signaling pathway, KRAS signaling activation, apoptosis, reactive oxygen species (ROS) pathway, androgen response, peroxisome, oxidative phosphorylation, and fatty acid, heme, bile acid, xenobiotic metabolism pathways (Figure 2c, Table S5). Whereas the special genes of cluster 2 in riced in 7 pathways, including mitotic spindle, glycolysis, G2M checkpoint, E2F target, Wnt-β/catenin signaling pathway, Hedgehog signaling pathway, angiogenesis (Figure 2d, Table S5).

TME consists of tumor cells, diverse immune and stromal cells, which have been reported to the significance in predicting patient outcomes and treatment efficacy.^54^ Therefore, we investigated the important biological pathways in TME, including epithelial-mesenchymal transition (EMT), angiogenesis, Wnt signaling pathway, and CD8 T effector, DNA damage repair, and nucleotide excision related pathways from previous research.^48^ And the results showed that the special genes of cluster 1 enriched in CD8 T effector, TLR signaling, TCR signaling, immune checkpoint, INF-γ, antigen processing machinery, CLR signaling, and EMT1 pathways. However, the special gene of cluster 2 enriched in Homologous recombination, Wnt signaling, DNA damage repair pathways (Figure 2e, Table S6). These results suggested that cluster 1 might elucidate high immunoreactivity, but cluster 2 might imply high immunosuppression in TME.

### Immune infiltration characterization in two m6A-related subclasses

**Figure 3.**
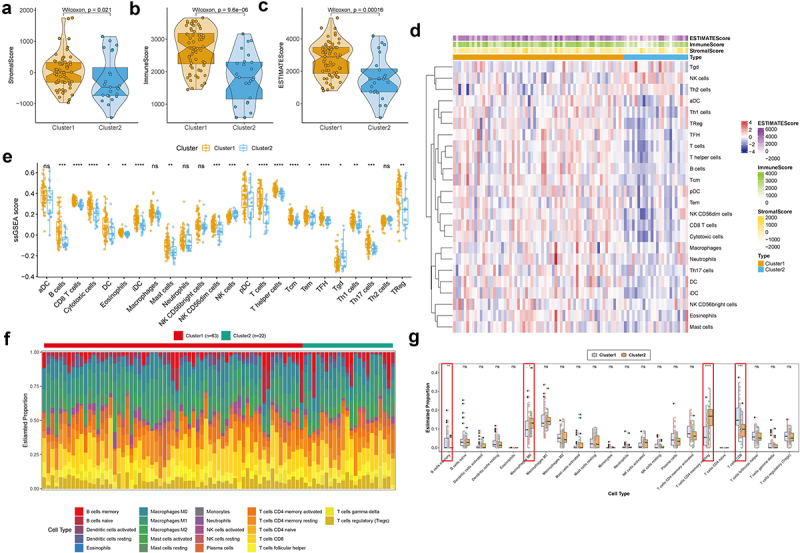
Immune infiltration characterization in two m6A-related subclasses a-c. Violin plot indicating stromal score, immune score and estimate score between two m6A-related subclasses. d. Heatmap showing the TME cell infiltrating between two m6A-related subclasses. e. Boxplot showing distinct TME cell infiltrating between two m6A-related subclasses. f. The staked abundance of immune cells between two m6A-related subclasses. g. Differences in immune cells between two m6A-related subclasses.

Based on previous analyses, we further explored the immune landscape of m6A-related subclasses in NPC. We obtained the ImmuneScore, StromalScore, and ESTIMATEScore of each sample, resulting in ImmuneScore and StromalScore being higher in cluster 1 than cluster 2 (Figure 3a–c, Table S7). We further used ssGSEA to identify the distinction of the infiltrating immune cells in two m6A-related subclasses. Twenty-four immune cell types were divided into two subclusters and shown in Figure 3d, e and Table S8, we observed that B cells, CD8 T cells, cytotoxic cells, dendritic cells (DCs), eosinophils, immature DCs (iDCs), mast cells, NKCD56dim cells, plasmacytoid DCs (pDCs), T cells, T helper cells, central memory T (Tcm) cells, effector memory T (Tem) cells, follicular helper T (TFH) cells, T help (Th) 1 cell, Th17 cells, Treg cells increased cluster 1 compared to cluster 2, whereas natural killer (NK) cells and Tgd cells elevated in cluster 2 than cluster 1. These results showed that there were significant differences in immune cells between two m6A-related subclasses, which suggested that m6A modification is associated with the immune landscape in NPC.

Also, the differences in the immune status of m6A-related subclasses in NPC was assessed by the CIBERSORT algorithm. The stacked abundance of 22 immune cells was showed in Figure 3f. As result, B cells memory and T cells CD8 were more enriched in cluster 1 than cluster 2, while Macrophage M0 and T cells CD4 memory resting were less enriched in cluster 1 than cluster 2 (Figure 3g, Table S8). The infiltration of the co-exist immune cells from two immune infiltration analysis, i.e. B cells memory (CD20 and CD27),^55^ T cells CD8 (CD4 and CD 8), Macrophage M0 (CD68) and T cells CD4 memory resting (CD4 and CD45RO)^56^ was detected by IHC in tumor and corresponding para-tumor tissues. The positive rate of CD4, CD45RO, and CD68 was higher, while the positive rate of CD20, CD27 and CD8 was lower in tumor tissues than those in corresponding non tumor tissues, which was coincident with the result of immune infiltration analysis (Figure S3).

### Correlation of the two m6A-related subclasses and the immunotherapy responses

**Figure 4.**
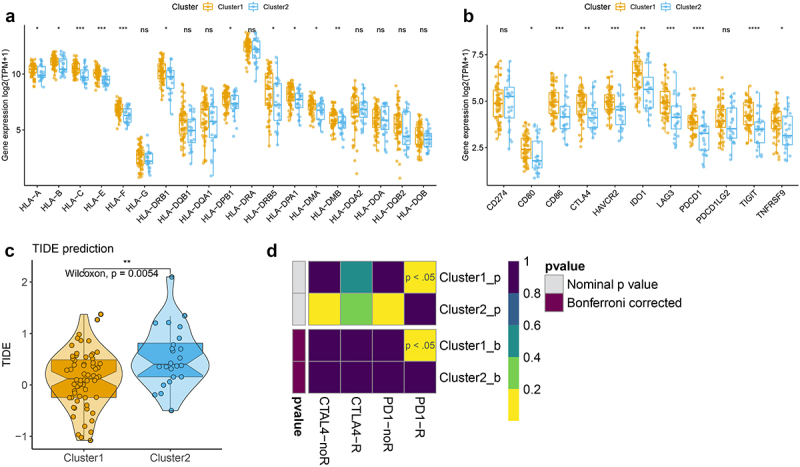
Correlation of the two m6A-related subclasses and the immunotherapy responses a. Boxplot indicating the distinct expression of HLA genes (HLA-A/B/C/E/F/G/DRB1/DQB1/DQA1/DPB1/DRA/DRB5/DPA1/DMA/DMB/DQA2/DOA/DQB2/DOB) between two m6A-related subclasses. b. Boxplot indicating the distinct expression of immune checkpoints (IDO1, PD-L1 (CD274), PD-L2 (PDCD1LG2), TIM-3 (HAVCR2), PD-1 (PDCD1), CTLA-4, CD80, CD86) between two m6A-related subclasses. c. Violin plot indicating TIDE score between two m6A-related subclasses. D. Submap matrix analysis showing response to checkpoint inhibitors PD-1 and CTLA4 in between two m6A-related subclasses.

According to the previous results, we speculated that cluster 1 was clustered as immune activation phenotype, and cluster 2 was clustered as immune suppression phenotype. Thus, we further test the expression of HLA family and immune checkpoints between two m6A-related subclasses, and the results indicated that HLA-A/B/C/E/F/DRB1/DPB1/DRB5/DPA1/DMA/DMB high expression in cluster 1 than cluster 2 (Figure 4a, Figure S4, Table S9), as well as the expression of CD80, CD86, CTLA4, HAVCR2, IDO1, LAG3, PDCD1, TIGIT, TNFTSF9 upregulated in cluster 1 compared to cluster 2 (Figure 4b, Table S10). We further detected the TIDE score and found a low TIDE score in cluster 1, but a high TIDE score in cluster 2 (Figure 4c, Table S11). We also compare the response to immunotherapy between two clusters by Submap, the results indicated that the patients in cluster 1 showed a better response to anit-PD1 and anti-CTLA4 treatment (Figure 4d). These findings suggested that the patients in cluster 1 may respond better to immunotherapy treatment.

### Correlation of the two m6A-related subclasses and chemotherapy response

**Figure 5.**
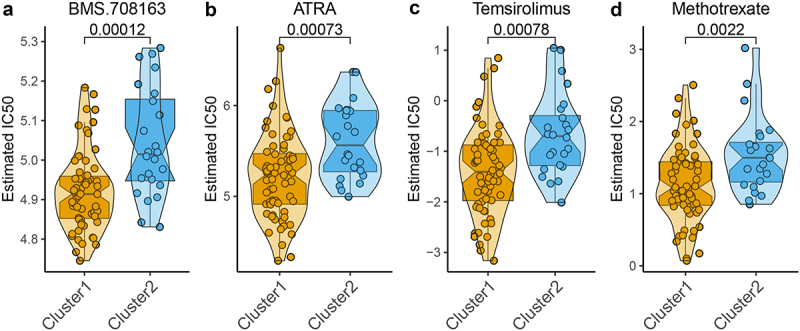
Correlation of the two m6A-related subclasses and chemotherapy response a-d. Violin plot showing IC50 value of BMS.708163, ATRA, temsirolimus, and methotrexate for each patient from two m6A-related subclasses.

Since m6A molecular patterns of NPC are associated with the response of treatment and clinical outcome, the relationship between m6A-related subclasses and chemotherapeutic efficacy was explored. We performed Ridge’s regression to predicate the sensitivity of each sample of two subclasses to 139 common chemotherapeutic drugs. The IC50 values were calculated and demonstrated that the patients in cluster 1 showed a better response to 17 chemotherapeutic drugs, including BMS.708163, ATRA, Temsirolimus, Methotrexate, BMS.536924, AKT inhibitor III, RDEA119, PF.02341066, DMOG, CI.1040, Bryostatin.1, AICAR, PD.0332991, Lenalidomide, NVP.TAE684, AZD6244, and LFM.A13 (Figure S3), especially BMS.708163, ATRA, Temsirolimus, Methotrexate (Figure 5). These findings indicated that m6A modification affected the chemotherapeutic outcome in NPC.

### Identification of the DEGs and their function enrichment in two m6A-related subclasses

**Figure 6.**
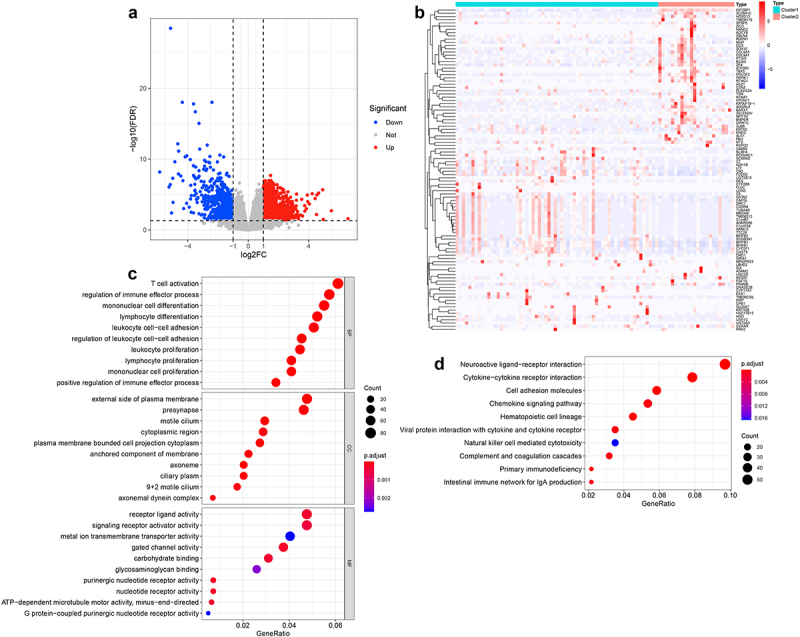
Identification of the DEGs and their function enrichment in two m6A-related subclasses a. Volcano plot showing the DEGs between two m6A-related subclasses. b. Heatmap showing the DEGs between two m6A-related subclasses. c. Bubble plot indicating the GO enrichment of DEGs between two m6A-related subclasses, including top 10 biological processes (BP), top 10 cellular components (CC), and top 10 molecular functions (MF) terms. d. Bubble plot indicating the KEGG pathways enrichment of DEGs between two m6A-related subclasses.

To further explore the potential regulatory mechanism of m6A modification in NPC, we identified 1558 DEGs (928 upregulated and 630 downregulated) between two m6A-related subclasses (Figure 6a, b, Table S12). And GO analysis indicated that the biological processes of those DEGs mainly involved in biological behaviors, such as immune cell T cell activation, regulation of immune effector process, mononuclear cell, lymphocyte, leukocyte, lymphocyte, mononuclear cell proliferation, leukocyte cell, or regulation of leukocyte cell–cell adhesion, positive regulation of immune effector process (Figure 6c, Table S13). And the CC terms enrichment included the external side of the plasma membrane, presynapse, motile cilium, cytoplasmic region, plasm membrane-bounded cell projection cytoplasm, the anchored component of membrane, axoneme, ciliary plasm, motile cilium, axonemal dynein complex (Figure 6c, Table S13). Besides, the molecular function of those DEGs involved in receptor-ligand, signaling receptor activator, metal ion transmembrane transporter, gate channel, purinergic nucleotide receptor, nucleotide receptor, and G-protein-coupled purinergic nucleotide receptor activity, carbohydrate and glycosaminoglycan binding, and ATP-dependent microtubule motor activity, minus-end-directed (Figure 6c, Table S13). Moreover, KEGG pathway enrichment analysis indicated that those DEGs significantly enriched in 16 pathways, most of them were immune-related pathways, such as hematopoietic cell lineage, cytokine-cytokine receptor interaction, viral protein interaction with cytokine and cytokine receptor, chemokine signaling pathway, complement and coagulation cascades, the intestinal immune network for IgA production, natural killer cell-mediated cytotoxicity, and other pathways, such as cell adhesion molecules, calcium signaling pathway, Metabolism of xenobiotics by cytochrome P450 (Figure 6d, Table S14). These results suggested that m6A modification affected tumor progression of NPC mainly through modulating immune-related pathways.

### Identification of m6A-related prognostic signature in NPC

We constructed the prognostic signature using univariate Cox and LASSO regression analyses to determine the prognostic value of m6A-related DEGs in the training set from the GSE102349 dataset. As shown in Table 4, 160 DEGs have been found associated with PFS. LASSO regression analysis was then

**Table 4.** Univariate Cox regression analysis of m6A-related DEGs associated to survival in GSE102349 dataset.

gene	KM	HR	HR.95 L	HR.95 H	Cox *p* value
IGF2BP1	0.012628	1.073979	1.015956	1.135317	0.011783
ADGRL3	0.044967	1.47551	1.207602	1.802855	0.000142
MLLT11	0.005147	1.026138	1.003228	1.049571	0.025111
RNF182	0.034559	1.368143	1.14517	1.634532	0.000554
EFR3B	0.009158	1.240523	1.096677	1.403237	0.000609
CD27	0.013334	0.963003	0.930569	0.996567	0.031029
NLRC5	0.007177	0.92927	0.866725	0.996329	0.039074
RASAL3	0.001762	0.936741	0.880532	0.996538	0.03847
LY9	0.011471	0.655851	0.430418	0.999354	0.04965
TBC1D10C	0.001546	0.930917	0.870911	0.995058	0.035231
CBARP	0.040803	2.11387	1.458998	3.062682	7.60E–05
PCED1B	0.013238	0.903792	0.826413	0.988416	0.026752
CD3D	0.010188	0.984679	0.971154	0.998392	0.028663
TRAF3IP3	0.010846	0.844529	0.721591	0.988411	0.035275
PTPN22	0.009157	0.859646	0.750116	0.985169	0.029642
ARHGAP15	0.001299	0.838939	0.705966	0.99696	0.046092
CEACAM21	0.010188	0.724025	0.531468	0.986349	0.040648
GPR174	0.001635	0.834773	0.700108	0.995341	0.04422
BIN2	0.001762	0.889249	0.811549	0.974389	0.011865
EVI2B	0.001635	0.976535	0.954131	0.999465	0.044949
CXCR3	0.005879	0.944605	0.901096	0.990214	0.017849
CD52	0.008718	0.990146	0.980922	0.999457	0.038099
GTSF1	0.006405	0.261762	0.089665	0.76417	0.014206
NLRC3	0.009157	0.866397	0.755719	0.993284	0.039725
EVI2A	0.0009	0.908202	0.842952	0.978503	0.011366
FCGR2B	0.029671	0.848706	0.735456	0.979395	0.024775
CYSLTR1	0.046598	0.640037	0.422069	0.970569	0.035677
MFNG	0.001546	0.917265	0.841962	0.999303	0.048164
FYB1	0.001762	0.93689	0.885425	0.991346	0.02373
RCOR2	0.040169	1.142094	1.061587	1.228706	0.000367
SLC6A15	0.022349	1.070061	1.013891	1.129343	0.013839
ST8SIA4	0.006405	0.813765	0.683501	0.968856	0.020589
CD247	0.010188	0.908558	0.835723	0.987741	0.024495
FAM78A	0.008718	0.870494	0.775386	0.977268	0.018799
SLA	0.009033	0.896627	0.822834	0.977038	0.012772
SCML4	0.001762	0.322926	0.119682	0.871317	0.025617
HERPUD1	0.030671	0.978259	0.95818	0.998759	0.037771
GMFG	0.008105	0.97444	0.95349	0.995851	0.019547
PLCB2	0.002763	0.876422	0.779654	0.9852	0.027122
RAB39B	0.001762	0.589769	0.348093	0.999237	0.049669
P2RY10	0.001438	0.911691	0.835201	0.995185	0.038649
ADA2	0.013948	0.955581	0.914979	0.997986	0.040268
SIDT1	0.001524	0.630736	0.417126	0.953737	0.028927
MAP2	0.024619	1.120266	1.054157	1.190521	0.000253
ABCD2	0.00234	0.323112	0.126893	0.822749	0.017832
CD48	0.001167	0.964389	0.931568	0.998367	0.040124
CD2	0.0096	0.982111	0.969128	0.995267	0.007843
PLD4	0.010578	0.846305	0.72025	0.994421	0.042565
CHRNB2	0.009166	1.369398	1.120744	1.67322	0.002105
S1PR4	0.001546	0.91596	0.842541	0.995777	0.039472
AGAP2	0.009157	0.783279	0.621824	0.986656	0.038076
CD3G	0.009157	0.889923	0.804293	0.98467	0.023868
FGD3	0.013916	0.88579	0.789157	0.994257	0.039619
GIMAP7	0.002888	0.973851	0.952377	0.99581	0.019856
CD226	0.044941	0.304647	0.10818	0.857925	0.024446
TLR7	0.036733	0.824277	0.686592	0.989573	0.038231
ANXA2R	0.001885	0.687824	0.481712	0.982128	0.039475
CRIP1	0.011447	0.532303	0.324141	0.874147	0.012723
POU3F2	0.00693	1.105457	1.04095	1.173961	0.001082
ATP8A2	0.041188	1.043686	1.009469	1.079062	0.011933
RAB33A	0.004695	0.856213	0.734482	0.998119	0.047254
CMTM7	0.002234	0.830569	0.724731	0.951864	0.007601
PATL2	0.001056	0.512255	0.312774	0.83896	0.007871
REEP2	0.009044	1.171318	1.090172	1.258505	1.58E–05
TBX21	0.011786	0.793671	0.631752	0.997089	0.047144
PLAAT3	0.002121	0.680803	0.530752	0.873274	0.002473
FCRL6	0.010564	0.290776	0.104801	0.806772	0.017676
KCNA3	0.044592	0.808268	0.661469	0.987646	0.037386
IGFLR1	0.012548	0.585373	0.366913	0.933904	0.024648
GFI1	0.001521	0.528727	0.342959	0.815119	0.003907
DIPK1C	0.004263	1.052991	1.017858	1.089337	0.002861
CTSW	0.008348	0.90993	0.842745	0.98247	0.015871
TBX20	0.004397	1.153943	1.024013	1.300358	0.018809
DPEP2	0.013901	0.489906	0.247826	0.968453	0.040155
CXCR6	0.006405	0.898834	0.836526	0.965783	0.003616
DLX2	0.032992	1.351225	1.011332	1.805352	0.041731
GIMAP1	0.017569	0.802687	0.655774	0.982512	0.033087
TFAP2A	0.014624	1.021693	1.007051	1.036547	0.003567
IL10RA	0.010188	0.931145	0.874014	0.99201	0.027225
SOWAHD	0.048003	0.757014	0.57474	0.997093	0.047628
GIMAP6	0.016687	0.937978	0.887876	0.990908	0.022251
VILL	0.046254	0.662976	0.484858	0.906529	0.010033
GIMAP4	0.016687	0.977523	0.958211	0.997225	0.025552
GAB3	0.041169	0.768891	0.609308	0.970269	0.026811
IDNK	0.000854	0.679495	0.484313	0.953339	0.025316
TMSB15A	0.031847	1.038879	1.018908	1.059241	0.000117
DLX1	0.04399	1.227529	1.023337	1.472463	0.02721
IQGAP2	0.001885	0.799359	0.645944	0.989211	0.039424
SLFN12L	0.001355	0.402002	0.19212	0.84117	0.015559
ZNF671	0.013948	0.718096	0.519043	0.993486	0.045563
CD38	0.009446	0.898639	0.819066	0.985943	0.023869
ABCB1	0.016002	0.345615	0.135974	0.87848	0.025603
TMEM273	0.000987	0.634623	0.454028	0.887052	0.007781
FAM178B	0.049025	1.221902	1.036315	1.440726	0.017109
CYP26A1	0.000815	1.026347	1.008689	1.044314	0.003314
HCLS1	0.011711	0.977532	0.955732	0.999829	0.048289
GPR65	0.001762	0.722405	0.565944	0.922121	0.009028
INPP4B	0.001668	0.562232	0.320168	0.987311	0.045025
GXYLT2	0.042667	1.104171	1.029779	1.183937	0.005361
MPEG1	0.011515	0.970184	0.944187	0.996897	0.028947
NINL	0.009193	1.062196	1.010069	1.117014	0.018764
TREML1	0.011579	0.35026	0.14914	0.822592	0.016028
KLF2	0.003663	0.88332	0.797791	0.978018	0.016952
PTPRN2	0.012737	0.534851	0.293531	0.974568	0.040942
HOXD13	0.029287	1.255992	1.112899	1.417483	0.000221
MFAP2	0.032649	1.018206	1.000677	1.036042	0.04171
CLIC5	0.011856	0.401561	0.164993	0.977323	0.044377
PCDHB14	0.017706	1.33013	1.133466	1.560916	0.000475
HCST	0.012009	0.91589	0.849545	0.987416	0.022018
CD1D	0.009602	0.483971	0.247629	0.945883	0.033779
RND2	0.031391	1.290796	1.028302	1.620295	0.027767
RASL10B	0.020422	1.917454	1.290184	2.849695	0.00128
RAC3	0.02889	1.055523	1.022867	1.089222	0.000752
SYCP2L	0.039063	1.589841	1.096184	2.305812	0.014522
ADRA1D	0.024166	1.052784	1.014903	1.092079	0.005938
KIF7	0.000934	1.129571	1.064332	1.198809	5.97E–05
FMO2	0.012725	0.75458	0.576407	0.987829	0.040455
SLC6A8	0.001899	1.00356	1.001685	1.005438	0.000196
ADGRB2	0.029333	1.052839	1.012843	1.094413	0.009166
HOXC13	0.002055	1.18416	1.050425	1.334922	0.0057
HOXD11	0.007764	1.402893	1.140134	1.726208	0.001377
SLC29A4	0.012205	1.152486	1.065132	1.247004	0.000417
SOWAHA	0.007358	1.219681	1.084304	1.371961	0.000939
LRP4	0.04516	1.021461	1.00508	1.038109	0.010044
ATP8A1	0.001121	0.792766	0.632608	0.993472	0.043711
HSH2D	0.013265	0.959393	0.921831	0.998484	0.041911
MAPK8IP2	0.006198	1.146456	1.04867	1.253361	0.002658
CLEC9A	0.008519	0.132406	0.035853	0.48898	0.002419
KLRD1	0.01313	0.075299	0.008979	0.631477	0.017142
C16orf54	0.016888	0.894327	0.809401	0.988163	0.028244
DSEL	0.04346	1.823329	1.368843	2.428713	4.02E–05
SH3GL2	0.000172	1.374435	1.033521	1.827803	0.028768
PDZD4	0.009421	1.059562	1.006381	1.115554	0.027661
DKK1	0.006721	1.004585	1.00104	1.008142	0.011197
SRSF12	0.015096	1.300295	1.136727	1.487399	0.000129
RGMA	0.014157	1.068853	1.020134	1.119898	0.005151
SYTL2	0.01592	0.49023	0.273577	0.878456	0.016602
CXCL1	0.018648	1.002412	1.001048	1.003778	0.000524
ID4	0.007478	1.039703	1.022184	1.057521	7.10E–06
LDHD	0.012326	0.102781	0.017315	0.61009	0.012288
FOLH1	0.037217	1.155669	1.041894	1.281867	0.006217
KMO	0.013441	0.504151	0.257532	0.986938	0.045681
DPYSL5	0.000904	1.193504	1.016091	1.401895	0.031211
TMIGD2	0.042842	0.608513	0.37178	0.995986	0.048159
DNAJC5B	0.009839	0.220927	0.058651	0.832183	0.025651
TUB	0.006556	1.126499	1.038824	1.221573	0.00396
TMEM108	0.011471	1.158237	1.072867	1.250401	0.00017
GPR50	0.014195	1.010617	1.002145	1.01916	0.01394
TAGLN3	0.027185	1.01759	1.007336	1.027948	0.00074
CTTNBP2	0.015065	1.039792	1.010092	1.070365	0.008312
C1GALT1C1L	0.041997	1.334447	1.146796	1.552803	0.00019
CACNG4	0.017501	1.024077	1.00925	1.039123	0.001387
RAET1L	0.046381	1.035038	1.013102	1.057449	0.001627
KIF1A	0.044196	1.13127	1.060399	1.206878	0.000187
PHYHD1	0.015863	0.263666	0.079617	0.873181	0.029115
SALL3	0.011066	1.149452	1.025139	1.288839	0.017073
PXDN	0.030061	1.012588	1.005183	1.020047	0.000836
BCHE	0.004569	1.173144	1.078425	1.276183	0.000201
SEMA6D	0.018727	1.01885	1.007344	1.030488	0.00127
IRX4	0.039418	1.089048	1.023365	1.158947	0.007196

performed to reduce the number of DEGs (Figure 7a, b). And REEP2, TMSB15A, DSEL, and ID4 were screed out and used to construct the risk model (Figure 7c). Then, the risk score of each sample in the training set, test set, and the whole set was calculated (Table S15–17), and all samples of each set were divided into a high-risk score and low-risk score groups, and the high-risk score associated to high mortality rate both in training, test, and whole sets (Figure 7d–f). Then, Kaplan-Meier curves were plotted with log-rank test and ROC curves to detect the sensitivity and specificity of the risk score to predicate PFS. Kaplan–Meier curves predicated the poor survival rate of patients with the high-risk score than patients with low-risk score (Figure 7g–i). Moreover, ROC curves showed high sensitivity and specificity of a risk score to predicate PFS of NPC patients, the AUC was 0.988, 0.879, 0.784 at 1, 2, 3-year in the training set, the AUC was 0.829, 0.802, 0.802 at 1, 2, 3-year in the test set, the AUC was 0.915, 0.861, 0.772 at 1, 2, 3-year in whole set (Figure 7j–l). These results indicated and encouraged the sensitivity and reliability of the risk model for prognostic predicting. We also collected the NPC samples to validate the expression of m6A-related prognostic signature (DSEL, ID4, REEP2, and TMSB15A) in NPC. As shown in Figure 8a, b, DSEL, ID4, REEP2, and TMSB15A were upregulated in the NPC samples compared with the non-tumor samples. However, the expression of DSEL, ID4, REEP2, and TMSB15A not related the tumor progression (Figure 8c). In addition, the Kaplan–Meier curves indicated that high expression of ID4 showed poor survival than low expression of ID4 in NPC (Figure 8d). These data indicated that DSEL, ID4, REEP2, and TMSB15A could be used as the biomarkers for NPC.

**Figure 7.**
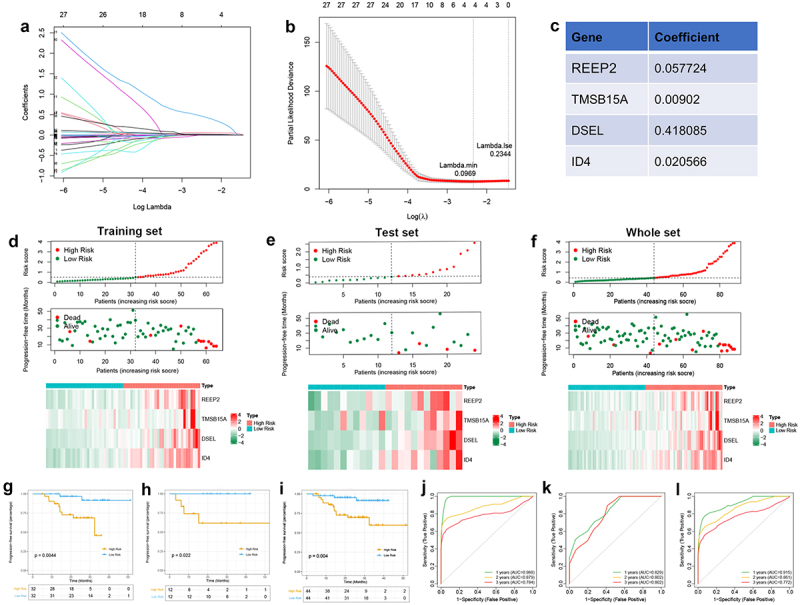
Identification of m6A-related prognostic signature in NPC A. Least absolute shrinkage and optimal LASSO coefficients of the 160 DEGs. B. Determination of the number of factors by the LASSO regression analysis. C. The sheet of showing the LASSO coefficients of four genes (REEP2, TMSB15A, DSEL, and ID4). D-F. The differences between high-risk score and low-risk score groups both in the training set, test set, and whole set. Upper: the distribution of risk core. Middle: the survival status of patients both in the high-risk score and low-risk score groups. Bottom: heatmap showing expression of m6A-related prognostic signature both in the high-risk score and low-risk score groups. G-I. Kaplan-Meier survival plot showing the PFS between high-risk score and low-risk score groups in the training set, test set, and the whole set. J-L. ROC curves indicate the 1-, 2-, 3-years survival of NPC patients both in the training set, test set, and whole set.

**Figure 8.**
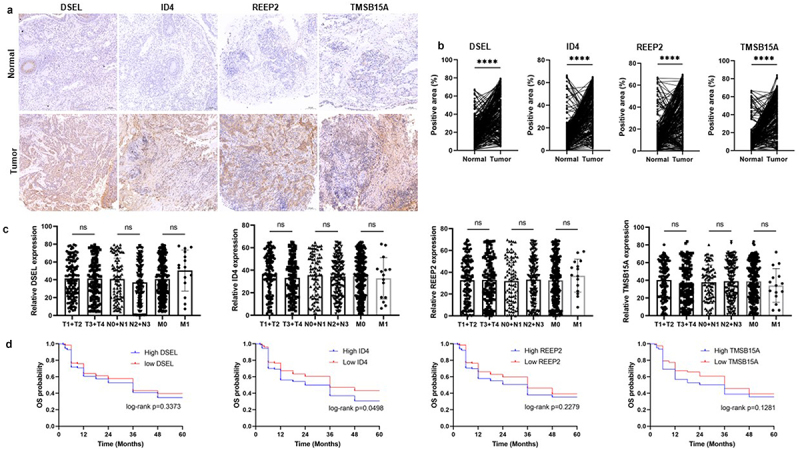
Validation of the expression of the DSEL, ID4, REEP2, and TMSB15A in NPC. a-b. IHC analyses of the levels of DSEL, ID4, REEP2, and TMSB15A in NPC samples (*n* = 255) compared with non-tumor samples (*n* = 255) (scale bars = 100 μm). c. Correlation between the levels of DSEL, ID4, REEP2, and TMSB15A and TNM stages. d. Kaplan-meier curves showing the OS of the NPC patients with high- and low-expression of DSEL, ID4, REEP2, and TMSB15A. ****, *p* < .0001.

### Development of a predicated model for NPC

**Figure 9.**
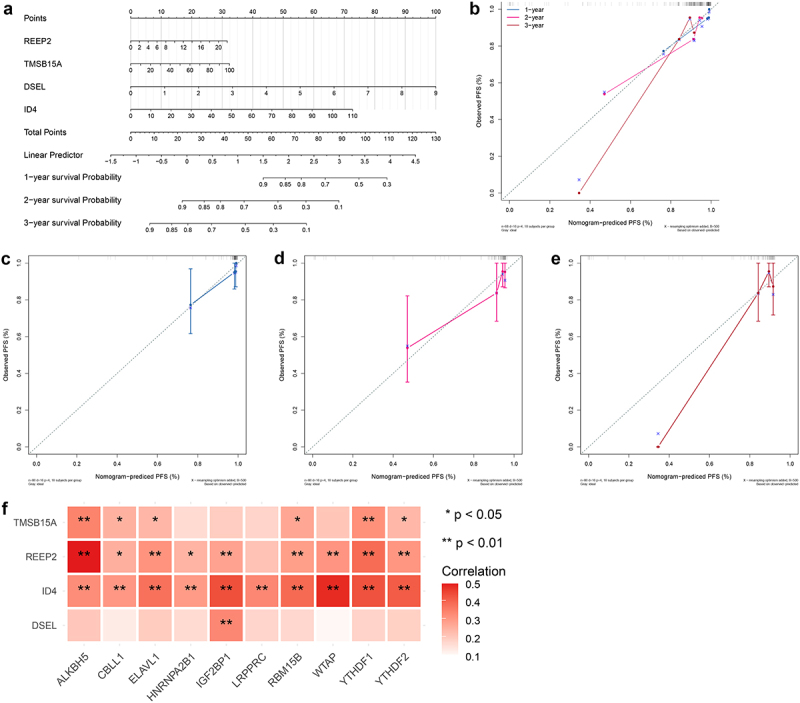
Development of a predicated model for NPC A. A predicating nomogram predicting PFS of NPC patients. B-E. Calibration plots assess the accuracy and specificity of the prognostic nomogram.F. Heatmap showing the correlation between prognostic signature and 10 m6A regulators.

To further validate the predictive accuracy prognostic signature, we developed a nomogram to predicate the survival probability at 1, 2, 3 years. As shown in Figure 9a, a high total score was calculated to predicate the poor survival probability at 1, 2, 3 years. And calibration curves were plotted to evaluate the accuracy and specificity of the prognostic nomogram, and the plots showed that the prediction lines of 1, 2, 3-year survival closed to the observation lines (Figure 9b–e), suggesting that the excellent accuracy of the prognostic nomogram. In addition, Pearson’s correlation analysis was performed to test the relationship between prognostic signature and m6A regulators, resulting in ID4 positively related to 10 m6A regulators, REEP2 positively related to 9 m6A regulators excepted LRPPRC, TMSB15A positively related to ALKBH5, CBLL1, ELAVL1, RBM15B, YTHDF1, YTHDF2, and DSEL positively related to IGF2BP1 (Figure 9f). These results indicated that m6A modification of prognostic signature might affect the clinical outcome of NPC patients.

### GSEA and TFs prediction

**Figure 10.**
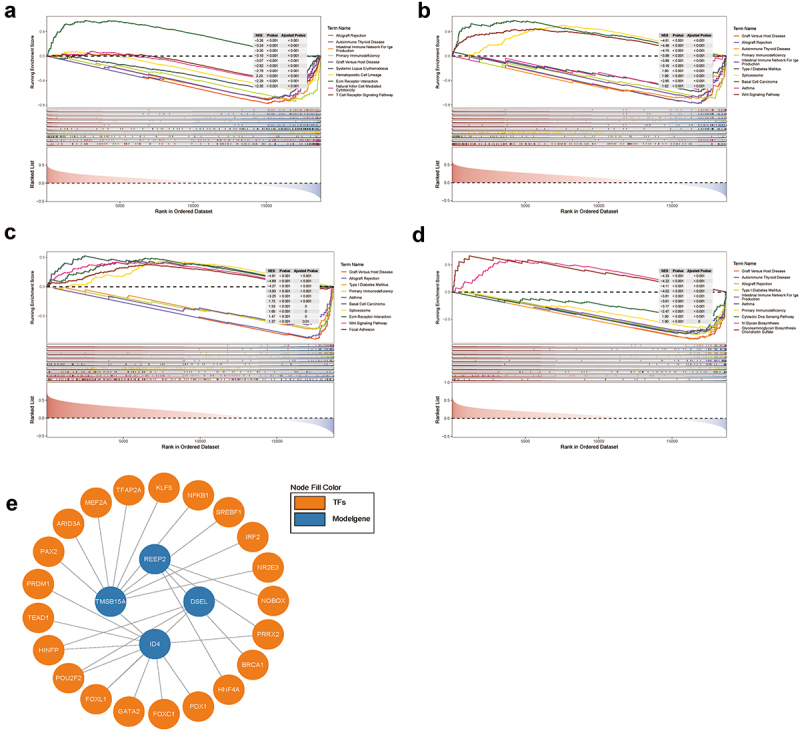
GSEA and transcription factor prediction of DSEL, ID4, REEP2, and TMSB15A a. TOP 10 KEGG enrichment of GSEA for DSEL, b.TOP 10 KEGG enrichment of GSEA for ID4, c.TOP 10 KEGG enrichment of GSEA for REEP2, d.TOP 10 KEGG enrichment of GSEA for TMSB15A, e. Transcription factor prediction of DSEL, ID4, REEP2, and TMSB15A.

To further investigate the signaling pathways and potential biological mechanisms for hub genes in NPC, GSEA was used. When threshold was set as padjust < 0.05 and |NES| > 1, DSEL was enriched in 49 KEGG items, with TOP 10 pathway as Allograft Rejection, Autoimmune Thyroid Disease, Intestinal Immune Network for IgA Production, Primary Immunodeficiency, Graft Versus Host Disease, Systemic Lupus Erythematosus, Hematopoietic Cell Lineage, ECM Receptor Interaction, Natural Killer Cell Mediated Cytotoxicity, T Cell Receptor Signaling Pathway (Figure 10a, Table 5, Table S19–20). ID4 was enriched in 21 KEGG items, with TOP 10 pathway as Graft versus host disease, Allograft rejection, Autoimmune thyroid disease, Primary immunodeficiency, Intestinal immune network for IgA production, Type I diabetes mellitus, Spliceosome, Basal cell carcinoma, Asthma, Wnt signaling pathway (Figure 10b, Table 6, Table S21–22). REEP2 was enriched in 12 items, with TOP 10 pathway as Graft Versus Host Disease, Allograft Rejection, Type I Diabetes Mellitus, Primary Immunodeficiency, Asthma, Basal Cell Carcinoma, Spliceosome, ECM Receptor Interaction, Wnt Signaling Pathway, Focal Adhesion (Figure 10c, Table 7, Table S23–24). TMSB15A was enriched in 18 items, with TOP 10 pathway as Graft Versus Host Disease, Autoimmune Thyroid Disease, Allograft Rejection, Type I Diabetes Mellitus, Intestinal Immune Network for IgA Production, Asthma, Primary Immunodeficiency, Cytosolic DNA Sensing Pathway, N Glycan Biosynthesis, Glycosaminoglycan Biosynthesis Chondroitin Sulfate (Figure 10d, Table 8, Table S25–26). Also, Transcription factors were predicted, a total of 21 TFs and 26 cooperative relationships between m6A-related prognostic signature and TFs was shown in Figure 10e, i.e. 4 for REEP2, 10 for ID4, 3 for DSEL, 8 for TMSB15A (Table S18).

**Table 5.** TOP 10 KEGG enrichment of GSEA for DSEL.

Description	setSize	enrichmentScore	NES	pvalue	p.adjust
KEGG_ALLOGRAFT_REJECTION	35	−0.77705	−3.26139	1.00E–10	2.61E–09
KEGG_AUTOIMMUNE_THYROID_DISEASE	50	−0.70576	−3.23748	1.00E–10	2.61E–09
KEGG_INTESTINAL_IMMUNE_NETWORK_FOR_IGA_PRODUCTION	46	−0.71065	−3.19615	1.00E–10	2.61E–09
KEGG_PRIMARY_IMMUNODEFICIENCY	35	−0.73977	−3.10493	1.00E–10	2.61E–09
KEGG_GRAFT_VERSUS_HOST_DISEASE	37	−0.72013	−3.06925	1.00E–10	2.61E–09
KEGG_SYSTEMIC_LUPUS_ERYTHEMATOSUS	129	−0.52359	−2.91846	1.00E–10	2.61E–09
KEGG_HEMATOPOIETIC_CELL_LINEAGE	84	−0.54644	−2.78867	1.00E–10	2.61E–09
KEGG_ECM_RECEPTOR_INTERACTION	83	0.58285	2.227741	7.82E–10	1.79E–08
KEGG_NATURAL_KILLER_CELL_MEDIATED_CYTOTOXICITY	131	−0.40807	−2.29007	1.40E–09	2.84E–08

**Table 6.** TOP 10 KEGG enrichment of GSEA for ID4.

Description	setSize	enrichmentScore	NES	pvalue	p.adjust
KEGG_GRAFT_VERSUS_HOST_DISEASE	37	−0.781109927	−4.608660761	1.00E–10	3.32E–09
KEGG_ALLOGRAFT_REJECTION	35	−0.778862515	−4.463503228	1.00E–10	3.32E–09
KEGG_AUTOIMMUNE_THYROID_DISEASE	50	−0.700140759	−4.148637492	1.00E–10	3.32E–09
KEGG_PRIMARY_IMMUNODEFICIENCY	35	−0.695521737	−3.985894125	1.00E–10	3.32E–09
KEGG_INTESTINAL_IMMUNE_NETWORK_FOR_IGA_PRODUCTION	46	−0.673964864	−3.891864232	1.00E–10	3.32E–09
KEGG_TYPE_I_DIABETES_MELLITUS	41	−0.587647574	−3.158684266	1.62E–10	4.48E–09
KEGG_SPLICEOSOME	126	0.502248333	1.796118137	2.74E–09	6.49E–08
KEGG_BASAL_CELL_CARCINOMA	55	0.584766766	1.987734988	9.61E–08	1.99E–06
KEGG_ASTHMA	28	−0.551205981	−2.853306438	1.18E–06	2.17E–05

**Table 7.** TOP 10 KEGG enrichment of GSEA for REEP2.

Description	setSize	enrichmentScore	NES	pvalue	p.adjust
KEGG_GRAFT_VERSUS_HOST_DISEASE	37	−0.893004479	−4.909084366	1.00E–10	3.90E–09
KEGG_ALLOGRAFT_REJECTION	35	−0.866988839	−4.693165585	1.00E–10	3.90E–09
KEGG_TYPE_I_DIABETES_MELLITUS	41	−0.72338884	−4.272212922	1.00E–10	3.90E–09
KEGG_PRIMARY_IMMUNODEFICIENCY	35	−0.726458503	−3.932449755	1.00E–10	3.90E–09
KEGG_ASTHMA	28	−0.652729273	−3.246932934	6.16E–09	1.92E–07
KEGG_BASAL_CELL_CARCINOMA	55	0.526014246	1.724377537	3.30E–05	0.000857107
KEGG_SPLICEOSOME	126	0.439907594	1.528457909	5.81E–05	0.001294707
KEGG_ECM_RECEPTOR_INTERACTION	83	0.48647439	1.651449842	9.53E–05	0.001858766
KEGG_WNT_SIGNALING_PATHWAY	150	0.416250379	1.465956475	0.000211989	0.003674482

**Table 8.** TOP 10 KEGG enrichment of GSEA for TMSB15A.

Description	setSize	enrichmentScore	NES	pvalue	p.adjust
KEGG_GRAFT_VERSUS_HOST_DISEASE	37	−0.864632197	−4.33279777	1.00E–10	2.72E–09
KEGG_AUTOIMMUNE_THYROID_DISEASE	50	−0.763448687	−4.217456164	1.00E–10	2.72E–09
KEGG_ALLOGRAFT_REJECTION	35	−0.86339686	−4.106595926	1.00E–10	2.72E–09
KEGG_TYPE_I_DIABETES_MELLITUS	41	−0.779596069	−4.018026653	1.00E–10	2.72E–09
KEGG_INTESTINAL_IMMUNE_NETWORK_FOR_IGA_PRODUCTION	46	−0.7303716	−3.811165931	1.00E–10	2.72E–09
KEGG_ASTHMA	28	−0.769302934	−3.60916266	1.00E–10	2.72E–09
KEGG_PRIMARY_IMMUNODEFICIENCY	35	−0.666101203	−3.168193694	3.05E–10	7.10E–09
KEGG_CYTOSOLIC_DNA_SENSING_PATHWAY	53	−0.448239504	−2.468193737	4.92E–07	1.00E–05
KEGG_N_GLYCAN_BIOSYNTHESIS	46	0.596512913	1.898460861	2.91E–06	5.27E–05

## Discussion

In this study, NPC patients were clustered as the two m6A-related molecular patterns based on 21 m6A regulators expression. And the characteristics of each cluster analysis indicated that cluster 1 associated immune activation and cluster 2 was associated with stromal activation. And the special genes of cluster 1 are involved in the immune-related pathways and metabolism pathways, and the special genes of cluster 2 are involved in the stromal-related pathways and tumorigenic pathways. The immune-related pathways involved in cluster 1 included the B cell receptor signaling pathway, T cell receptor signaling pathway, chemokine signaling pathway, natural killer cell-mediated cytotoxicity, and Toll-like receptor signaling pathway, antigen processing, and presentation. B and T cells are the key players of the adaptive immune system and are recognized and activated by the B cell receptor (BCR) and T cell receptor (TCR).^57^ BCR acts as a key role at multiple checkpoints of B cell biology,^58^ and the BCR signaling pathway exerts a crucial role in normal B cell development and adaptive immunity, targeting BCR signaling may have anticancer activity beyond B cell malignancies.^59^ TCR signaling pathway plays a central role in the control of T cell differentiation, homeostasis, and function, which regulate immune homeostasis and autoimmunity.^60–63^ Additionally, chemokine signaling pathway, natural killer cell-mediated cytotoxicity, and toll-like receptor signaling pathway, antigen processing, and presentation work in TME by trafficking and modulating immune cell proliferation, migration, activation, differentiation, and homing.^64–67^ Those immune activation-related pathways enriched in cluster 1, elucidate high immunoreactivity in cluster 1. Nevertheless, the special genes of cluster 2 enriched in stromal-related pathways, such as ECM receptor interaction, Wnt signaling pathway, adheres junction, Hedgehog signaling pathway. ECM is a noncellular component of TME stromal that forms as a scaffold in the tumor and responds to promoting tumor aggression.^68^ The Wnt signaling pathway is a high conserved signaling pathway that involves embryonic, organ development, and cancer progression.^69^ Wnt signaling activation is associated with stromal cell activation, such as cancer stem cells (CSCs) and cancer-associated fibroblasts (CAFs).^70–72^ Hedgehog (Hh) signaling pathway also acts as an essential role in maintaining CSC self-renewal and tumor progression, targeted treatment of Hh signaling pathway emerges the novel therapeutic strategy in a wide range of solid cancers.^73,74^ Therefore, we speculated that cluster 2 of NPC patients might associate with antitumor immunosuppress.

Numerous pieces of evidence have highlighted that m6A modification exerts an indispensable role in regulating the immune system by maintaining the naïve and pluripotent status of immune cells.^75^ Crosstalk between m6A modification and the immune system has the great significance to discover novel pathogenic mechanisms and to conduct promising therapeutic targets for tumors.^76^ M6A modification involvement in immune modulation in tumors includes regulating innate and adaptive immune cells^77^ and anti-tumor immunity.^78^ Therefore, we further investigated the immune landscape of m6A-related classes. And the results are consistent with the previous finding, B cells memory and T cells CD8 were more enriched in cluster 1 than cluster 2, while Macrophage M0 and T cells CD4 memory resting were less enriched in cluster 1 than cluster 2. ESTIMATE algorithm estimated ImmuneScore, StromalScore, and ESTIMATEScore of each sample, and results indicated that high ImmuneScore and StromalScore of cluster 1 than cluster 2. As known to us, the immune system can be recognized as an innate and adaptive immune system, that contributes to recognize and remove foreign pathogens and tumors.^79^ Adaptive immunity mainly includes T and B lymphocytes, which harbor plenty of TCRs and BCRs, that respond to different antigens in the pathological process.^80^ As expected, the immunotherapy response exploring indicated that the patients in the cluster exhibited a better response to anti-PD1 and anti-CTLA4 treatment. Additionally, we also found that the patients in cluster 1 were more sensitive to BMS.708163, ATRA, temsirolimus, and methotrexate. BMS.708163 is a gamma-secretase inhibitor and exerts antitumor effects in lung cancer by reversing EGFR inhibitor resistance,^81^ but it currently has not been reported for NPC treatment. ATRA is an active metabolite of vitamin A and used as an effective antitumor in acute promyelocytic leukemia,^82^ And emerges the antitumor effects in other tumors by a combination of ATRA with other agents, including chemotherapy, epigenetic modifiers, and others.^83^ ATRA also has been demonstrated that exert the antitumor effects on NPC.^84^ Temsirolimus is an mTOR inhibitor widely used in renal cell carcinoma and achieves good efficacy in the therapeutic course,^85^ it can rarely use in NPC treatment. Methotrexate (MTX) is widely used for antitumor therapy in a variety of childhood and adult cancers,^86^ it has been successfully treated NPC using an effective delivery method.^87^

The DEGs between two m6A-related subclasses were identified and the mechanism analysis indicated that DEGs mainly enriched immune-related pathways, suggesting that m6A modification regulated tumor progression by modulating immune-related pathways. The m6A-related prognostic signature trained in the GEO dataset was included REEP2, TMSB15A, DSEL, and ID4. The prognostic risk model and predicated nomogram for NPC were construed to estimate the prognosis of NPC.

## Conclusion

Taken together, our study demonstrated the significance of m6A modification in NPC by regulating TME. M6A modification patterns caused the heterogeneity and complexity of individual TME, resulting in different responses to therapy. Our findings contributed to our understanding of the pathogenic mechanisms of TME cell infiltrating and provided promising therapeutic targets for NPC. Although our research revealed the significance for understating and conducting treatment strategy of NPC, limitation of this study is small size of the NPC samples to validate the biomarkers, in the subsequent analyses, we will collect the larger NPC samples with corresponding clinical information to demonstrate our results.

## Supplementary Material

Supplemental Material

## Data Availability

All data in this study can be obtained from the Gene Expression Omnibus (GEO, https://www.ncbi.nlm.nih.gov/geo/). The analytical methods can be found in the methods section. R scripts written for performing analyses in this study are shared via GitHub (https://github.com/DrWangfeng/nasopharyngeal-carcinoma). And the supplementary files have been shared via GitHub (https://github.com/DrWangfeng/Supplementary-files.git).

## References

[1] Chen YP, Chan ATC, Le Q-T, Blanchard P, Sun Y, Ma J. Nasopharyngeal carcinoma. Lancet. 2019;394(10192):64–21. doi:10.1016/S0140-6736(19)30956-0. 31178151

[2] Luo W, Yao K, Busson P. Molecular characterization and clinical implications of spindle cells in nasopharyngeal carcinoma: a novel molecule-morphology model of tumor progression proposed. PloS One. 2013;8(12):e83135. doi:10.1371/journal.pone.0083135. 24349446 PMC3861507

[3] Bray F, Ferlay J, Soerjomataram I, Siegel RL, Torre LA, Jemal A. Global cancer statistics 2018: GLOBOCAN estimates of incidence and mortality worldwide for 36 cancers in 185 countries. CA Cancer J Clin. 2018;68(6):394–424. doi:10.3322/caac.21492. 30207593

[4] Ferlay J, Lam F, Colombet M, Mery L, Piñeros M, Znaor A, Soerjomataram I, Bray F. Global cancer observatory: cancer Today. Lyon, France: International Agency for Research on Cancer; 2020. https://gco.iarc.fr/today.

[5] Ferlay J, Colombet M, Soerjomataram I, Mathers C, Parkin DM, Piñeros M, Znaor A, Bray F. Estimating the global cancer incidence and mortality in 2018: GLOBOCAN sources and methods. Int J Cancer. 2019;144(8):1941–1953. doi:10.1002/ijc.31937. 30350310

[6] Wei KR, Zheng R-S, Zhang S-W, Liang Z-H, Li Z-M, Chen W-Q. Nasopharyngeal carcinoma incidence and mortality in China, 2013. Chin J Cancer. 2017;36(1):90. doi:10.1186/s40880-017-0257-9. 29122009 PMC5679327

[7] Lam WKJ, Chan JYK. Recent advances in the management of nasopharyngeal carcinoma. F1000Res. 2018. 7:1829. doi:10.12688/f1000research.15066.1. PMC6249636 30519454

[8] Sun XS, Li X-Y, Chen Q-Y, Tang L-Q, Mai H-Q. Future of radiotherapy in nasopharyngeal carcinoma. Br J Radiol. 2019;92(1102):20190209. doi:10.1259/bjr.20190209. 31265322 PMC6774595

[9] Zhang Y, Chen L, Hu G-Q, Zhang N, Zhu X-D, Yang K-Y, Jin F, Shi M, Chen Y-P, Hu W-H, et al. Gemcitabine and Cisplatin Induction Chemotherapy in nasopharyngeal carcinoma. N Engl J Med. 2019; 381(12):1124–1135. doi:10.1056/NEJMoa1905287. 31150573

[10] Lee AWM, Ng WT, Chan JYW, Corry J, Mäkitie A, Mendenhall WM, Rinaldo A, Rodrigo JP, Saba NF, Strojan P, et al. Management of locally recurrent nasopharyngeal carcinoma. Cancer Treat Rev. 2019; 79:101890. doi:10.1016/j.ctrv.2019.101890. 31470314

[11] Suárez C, Rodrigo JP, Rinaldo A, Langendijk JA, Shaha AR, Ferlito A. Current treatment options for recurrent nasopharyngeal cancer. Eur Arch Otorhinolaryngol. 2010;267(12):1811–1824. doi:10.1007/s00405-010-1385-x. 20865269 PMC2966947

[12] Lee HM, et al. 2019. Current perspectives on nasopharyngeal carcinoma. Adv Exp Med Biol. 1164:11–34. 31576537 10.1007/978-3-030-22254-3_2

[13] Campion NJ, Ally M, Jank BJ, Ahmed J, Alusi G. The molecular march of primary and recurrent nasopharyngeal carcinoma. Oncogene. 2021;40(10):1757–1774. doi:10.1038/s41388-020-01631-2. 33479496

[14] Hinshaw DC, Shevde LA. The tumor microenvironment innately modulates cancer progression. Cancer Res. 2019;79(18):4557–4566. doi:10.1158/0008-5472.CAN-18-3962. 31350295 PMC6744958

[15] Ding C, Shan Z, Li M, Chen H, Li X, Jin Z. Characterization of the fatty acid metabolism in colorectal cancer to guide clinical therapy. Mol Ther Oncolytics. 2021. 20:532–544. doi:10.1016/j.omto.2021.02.010. 33738339 PMC7941088

[16] Tao J, Yang G, Zhou W, Qiu J, Chen G, Luo W, Zhao F, You L, Zheng L, Zhang T, et al. Targeting hypoxic tumor microenvironment in pancreatic cancer. J Hematol Oncol. 2021; 14(1):14. doi:10.1186/s13045-020-01030-w. 33436044 PMC7805044

[17] Hanahan D, Weinberg RA. Hallmarks of cancer: the next generation. Cell. 2011;144(5):646–674. doi:10.1016/j.cell.2011.02.013. 21376230

[18] Barker HE, Paget JTE, Khan AA, Harrington KJ. The tumour microenvironment after radiotherapy: mechanisms of resistance and recurrence. Nat Rev Cancer. 2015;15(7):409–425. doi:10.1038/nrc3958. 26105538 PMC4896389

[19] Da Ros M, De Gregorio V, Iorio A, Giunti L, Guidi M, de Martino M, Genitori L, Sardi I. Glioblastoma Chemoresistance: the Double Play by microenvironment and blood-brain barrier. Int J Mol Sci. 2018;19(10):19(10. doi:10.3390/ijms19102879. PMC6213072 30248992

[20] Tauriello DVF, Palomo-Ponce S, Stork D, Berenguer-Llergo A, Badia-Ramentol J, Iglesias M, Sevillano M, Ibiza S, Cañellas A, Hernando-Momblona X, et al. TGFβ drives immune evasion in genetically reconstituted colon cancer metastasis. Nature. 2018; 554(7693):538–543. doi:10.1038/nature25492. 29443964

[21] Madden EC, Gorman AM, Logue SE, Samali A. Tumour cell secretome in chemoresistance and tumour recurrence. Trends Cancer. 2020;6(6):489–505. doi:10.1016/j.trecan.2020.02.020. 32460003

[22] Ren B, Cui M, Yang G, Wang H, Feng M, You L, Zhao Y. Tumor microenvironment participates in metastasis of pancreatic cancer. Mol Cancer. 2018;17(1):108. doi:10.1186/s12943-018-0858-1. 30060755 PMC6065152

[23] Gong L, Kwong DLW, Dai W, Wu P, Wang Y, Lee AWM, Guan X-Y. The stromal and immune landscape of nasopharyngeal Carcinoma and its implications for precision medicine targeting the tumor microenvironment. Front Oncol. 2021. 11:744889. doi:10.3389/fonc.2021.744889. 34568077 PMC8462296

[24] Liu H, Tang L, Li Y, Xie W, Zhang L, Tang H, Xiao T, Yang H, Gu W, Wang H, et al. Nasopharyngeal carcinoma: current views on the tumor microenvironment’s impact on drug resistance and clinical outcomes. Mol Cancer. 2024; 23(1):20. doi:10.1186/s12943-023-01928-2. 38254110 PMC10802008

[25] Zhang L, Hou C, Chen C, Guo Y, Yuan W, Yin D, Liu J, Sun Z. The role of N(6)-methyladenosine (m(6)A) modification in the regulation of circRnas. Mol Cancer. 2020;19(1):105. doi:10.1186/s12943-020-01224-3. 32522202 PMC7285594

[26] Yang Y, Hsu PJ, Chen Y-S, Yang Y-G. Dynamic transcriptomic m(6)A decoration: writers, erasers, readers and functions in RNA metabolism. Cell Res. 2018;28(6):616–624. doi:10.1038/s41422-018-0040-8. 29789545 PMC5993786

[27] Meyer KD, Jaffrey SR. The dynamic epitranscriptome: N6-methyladenosine and gene expression control. Nat Rev Mol Cell Biol. 2014;15(5):313–326. doi:10.1038/nrm3785. 24713629 PMC4393108

[28] Lin Z, Niu Y, Wan A, Chen D, Liang H, Chen X, Sun L, Zhan S, Chen L, Cheng C, et al. RNA m 6 a methylation regulates sorafenib resistance in liver cancer through FOXO 3-mediated autophagy. EMBO J. 2020; 39(12):e103181. doi:10.15252/embj.2019103181. 32368828 PMC7298296

[29] Wang L, Hui H, Agrawal K, Kang Y, Li N, Tang R, Yuan J, Rana TM. M 6 a RNA methyltransferases METTL3/14 regulate immune responses to anti-PD-1 therapy. EMBO J. 2020;39(20):e104514. doi:10.15252/embj.2020104514. 32964498 PMC7560214

[30] Xu S, Tang L, Dai G, Luo C, Liu Z. Expression of m6A regulators correlated with immune microenvironment predicts therapeutic efficacy and prognosis in gliomas. Front Cell Dev Biol. 2020. 8:594112. doi:10.3389/fcell.2020.594112. 33240891 PMC7683617

[31] Li N, Kang Y, Wang L, Huff S, Tang R, Hui H, Agrawal K, Gonzalez GM, Wang Y, Patel SP, et al. ALKBH5 regulates anti–PD-1 therapy response by modulating lactate and suppressive immune cell accumulation in tumor microenvironment. Proc Natl Acad Sci U S A. 2020; 117(33):20159–20170. doi:10.1073/pnas.1918986117. 32747553 PMC7443867

[32] Zheng ZQ, Li Z-X, Zhou G-Q, Lin L, Zhang L-L, Lv J-W, Huang X-D, Liu R-Q, Chen F, He X-J, et al. Long noncoding RNA FAM225A promotes nasopharyngeal carcinoma tumorigenesis and metastasis by acting as ceRNA to sponge miR-590-3p/miR-1275 and upregulate ITGB3. Cancer Res. 2019; 79(18):4612–4626. doi:10.1158/0008-5472.CAN-19-0799. 31331909

[33] Li ZX, Zheng Z-Q, Yang P-Y, Lin L, Zhou G-Q, Lv J-W, Zhang L-L, Chen F, Li Y-Q, Wu C-F, et al. WTAP-mediated m(6)A modification of lncRNA DIAPH1-AS1 enhances its stability to facilitate nasopharyngeal carcinoma growth and metastasis. Cell Death Differ. 2022; 29(6):1137–1151. doi:10.1038/s41418-021-00905-w. 34999731 PMC9177844

[34] Zhao BS, Roundtree IA, He C. Post-transcriptional gene regulation by mRNA modifications. Nat Rev Mol Cell Biol. 2017;18(1):31–42. doi:10.1038/nrm.2016.132. 27808276 PMC5167638

[35] Zhu H, Jia X, Wang Y, Song Z, Wang N, Yang Y, Shi X. M6A classification combined with tumor microenvironment immune characteristics analysis of bladder cancer. Front Oncol. 2021. 11:714267. doi:10.3389/fonc.2021.714267. 34604051 PMC8479184

[36] Zhang B, Wu Q, Li B, Wang D, Wang L, Zhou YL. m6A regulator-mediated methylation modification patterns and tumor microenvironment infiltration characterization in gastric cancer. Mol Cancer. 2020;19(1):53. doi:10.1186/s12943-020-01170-0. 32164750 PMC7066851

[37] Li M, Zha X, Wang S. The role of N6-methyladenosine mRNA in the tumor microenvironment. Biochim Biophys Acta Rev Cancer. 2021;1875(2):188522. doi:10.1016/j.bbcan.2021.188522. 33545295

[38] Quan C, Belaydi O, Hu J, Li H, Yu A, Liu P, Yi Z, Qiu D, Ren W, Ma H, et al. N(6)-methyladenosine in cancer immunotherapy: an undervalued therapeutic target. Front Immunol. 2021; 12:697026. doi:10.3389/fimmu.2021.697026. 34526985 PMC8436617

[39] Zhang P, He Q, Lei Y, Li Y, Wen X, Hong M, Zhang J, Ren X, Wang Y, Yang X, et al. m(6)A-mediated ZNF750 repression facilitates nasopharyngeal carcinoma progression. Cell Death Disease. 2018; 9(12):1169. doi:10.1038/s41419-018-1224-3. 30518868 PMC6281568

[40] Meng QZ, Cong C-H, Li X-J, Zhu F, Zhao X, Chen F-W. METTL3 promotes the progression of nasopharyngeal carcinoma through mediating M6A modification of EZH2. Eur Rev Med Pharmacol Sci. 2020;24(8):4328–4336. doi:10.26355/eurrev_202004_21014. 32373970

[41] He JJ, Li Z, Rong Z-X, Gao J, Mu Y, Guan Y-D, Ren X-X, Zi Y-Y, Liu L-Y, Fan Q, et al. m(6)A Reader YTHDC2 promotes radiotherapy resistance of nasopharyngeal carcinoma via activating IGF1R/AKT/S6 signaling axis. Front Oncol. 2020; 10:1166. doi:10.3389/fonc.2020.01166. 32850334 PMC7411471

[42] Zhang L, MacIsaac KD, Zhou T, Huang P-Y, Xin C, Dobson JR, Yu K, Chiang DY, Fan Y, Pelletier M, et al. Genomic analysis of nasopharyngeal carcinoma reveals TME-Based subtypes. Mol Cancer Res. 2017; 15(12):1722–1732. doi:10.1158/1541-7786.MCR-17-0134. 28851814

[43] Bao YN, Cao X, Luo D, Sun R, Peng L, Wang L, Yan Y, Zheng L, Xie P, Cao Y, et al. Urokinase-type plasminogen activator receptor signaling is critical in nasopharyngeal carcinoma cell growth and metastasis. Cell Cycle. 2014; 13(12):1958–1969. doi:10.4161/cc.28921. 24763226 PMC4111759

[44] Wilkerson MD, Hayes DN. ConsensusClusterPlus: a class discovery tool with confidence assessments and item tracking. Bioinformatics. 2010;26(12):1572–1573. doi:10.1093/bioinformatics/btq170. 20427518 PMC2881355

[45] Spiwok V, Kříž P. Time-Lagged t-distributed stochastic neighbor embedding (t-SNE) of molecular simulation trajectories. Front Mol Biosci. 2020. 7:132. doi:10.3389/fmolb.2020.00132. 32714941 PMC7344294

[46] Hoshida Y, Brunet J-P, Tamayo P, Golub TR, Mesirov JP. Subclass mapping: identifying common subtypes in independent disease data sets. PloS One. 2007;2(11):e1195. doi:10.1371/journal.pone.0001195. 18030330 PMC2065909

[47] Hänzelmann S, Castelo R, Guinney J. GSVA: gene set variation analysis for microarray and RNA-seq data. BMC Bioinf. 2013;14(1):7. doi:10.1186/1471-2105-14-7. PMC3618321 23323831

[48] Mariathasan S, Turley SJ, Nickles D, Castiglioni A, Yuen K, Wang Y, Kadel III EE, Koeppen H, Astarita JL, Cubas R, et al. TGFβ attenuates tumour response to PD-L1 blockade by contributing to exclusion of T cells. Nature. 2018; 554(7693):544–548. doi:10.1038/nature25501. 29443960 PMC6028240

[49] Yoshihara K, Shahmoradgoli M, Martínez E, Vegesna R, Kim H, Torres-Garcia W, Treviño V, Shen H, Laird PW, Levine DA, et al. Inferring tumour purity and stromal and immune cell admixture from expression data. Nat Commun. 2013; 4(1):2612. doi:10.1038/ncomms3612. 24113773 PMC3826632

[50] Subramanian A, Tamayo P, Mootha VK, Mukherjee S, Ebert BL, Gillette MA, Paulovich A, Pomeroy SL, Golub TR, Lander ES, et al. Gene set enrichment analysis: a knowledge-based approach for interpreting genome-wide expression profiles. Proc Natl Acad Sci U S A. 2005; 102(43):15545–15550. doi:10.1073/pnas.0506580102. 16199517 PMC1239896

[51] Bindea G, Mlecnik B, Tosolini M, Kirilovsky A, Waldner M, Obenauf A, Angell H, Fredriksen T, Lafontaine L, Berger A, et al. Spatiotemporal dynamics of intratumoral immune cells reveal the immune landscape in human cancer. Immunity. 2013; 39(4):782–795. doi:10.1016/j.immuni.2013.10.003. 24138885

[52] Jiang P, Gu S, Pan D, Fu J, Sahu A, Hu X, Li Z, Traugh N, Bu X, Li B, et al. Signatures of T cell dysfunction and exclusion predict cancer immunotherapy response. Nat Med. 2018; 24(10):1550–1558. doi:10.1038/s41591-018-0136-1. 30127393 PMC6487502

[53] Lu X, Jiang L, Zhang L, Zhu Y, Hu W, Wang J, Ruan X, Xu Z, Meng X, Gao J, et al. Immune signature-based subtypes of cervical squamous cell carcinoma tightly associated with human papillomavirus type 16 expression. Molecul Featur Clin Outcome. Neoplasia. 2019; 21(6):591–601. doi:10.1016/j.neo.2019.04.003. PMC6658934 31055200

[54] Zeng D, Li M, Zhou R, Zhang J, Sun H, Shi M, Bin J, Liao Y, Rao J, Liao W. Tumor microenvironment characterization in gastric cancer identifies prognostic and immunotherapeutically relevant gene signatures. Cancer Immunol Res. 2019;7(5):737–750. doi:10.1158/2326-6066.CIR-18-0436. 30842092

[55] Zhang Z, Ma L, Goswami S, Ma J, Zheng B, Duan M, Liu L, Zhang L, Shi J, Dong L, et al. Landscape of infiltrating B cells and their clinical significance in human hepatocellular carcinoma. Oncoimmunology. 2019; 8(4):e1571388. doi:10.1080/2162402X.2019.1571388. 30906667 PMC6422393

[56] Wu J, Zhang T, Xiong H, Zeng L, Wang Z, Peng Y, Chen W, Hu X, Su T. Tumor-infiltrating CD4(+) central memory T cells correlated with favorable prognosis in oral squamous cell carcinoma. J Inflamm Res. 2022. 15:141–152. doi:10.2147/JIR.S343432. 35035226 PMC8754505

[57] Fiala GJ, Minguet S. Caveolin-1: the unnoticed player in TCR and BCR signaling. Adv Immunol. 2018;137:83–133. 29455848 10.1016/bs.ai.2017.12.002

[58] Tanaka S, Baba Y. B Cell Receptor Signaling. Adv Exp Med Biol. 2020;1254:23–36. 32323266 10.1007/978-981-15-3532-1_2

[59] Burger JA, Wiestner A. Targeting B cell receptor signalling in cancer: preclinical and clinical advances. Nat Rev Cancer. 2018;18(3):148–167. doi:10.1038/nrc.2017.121. 29348577

[60] Courtney AH, Lo WL, Weiss A. TCR signaling: mechanisms of initiation and propagation. Trends Biochem Sci. 2018;43(2):108–123. doi:10.1016/j.tibs.2017.11.008. 29269020 PMC5801066

[61] Smith-Garvin JE, Koretzky GA, Jordan MS. T cell activation. Annu Rev Immunol. 2009;27(1):591–619. doi:10.1146/annurev.immunol.021908.132706. 19132916 PMC2740335

[62] Li MO, Rudensky AY. T cell receptor signalling in the control of regulatory T cell differentiation and function. Nat Rev Immunol. 2016;16(4):220–233. doi:10.1038/nri.2016.26. 27026074 PMC4968889

[63] Wing JB, Tanaka A, Sakaguchi S. Human FOXP3(+) regulatory T cell heterogeneity and function in autoimmunity and cancer. Immunity. 2019;50(2):302–316. doi:10.1016/j.immuni.2019.01.020. 30784578

[64] Zhou W, Guo S, Liu M, Burow ME, Wang G. Targeting CXCL12/CXCR4 axis in tumor immunotherapy. Curr Med Chem. 2019;26(17):3026–3041. doi:10.2174/0929867324666170830111531. 28875842 PMC5949083

[65] Prager I, Watzl C. Mechanisms of natural killer cell-mediated cellular cytotoxicity. J Leukoc Biol. 2019;105(6):1319–1329. doi:10.1002/JLB.MR0718-269R. 31107565

[66] Wang Y, Zhang S, Li H, Wang H, Zhang T, Hutchinson MR, Yin H, Wang X. Small-molecule modulators of toll-like receptors. Acc Chem Res. 2020;53(5):1046–1055. doi:10.1021/acs.accounts.9b00631. 32233400

[67] Wang S, He Z, Wang X, Li H, Liu X-S. Antigen presentation and tumor immunogenicity in cancer immunotherapy response prediction. Elife. 2019. 8:8. doi:10.7554/eLife.49020. PMC6879305 31767055

[68] Najafi M, Farhood B, Mortezaee K. Extracellular matrix (ECM) stiffness and degradation as cancer drivers. J Cell Biochem. 2019;120(3):2782–2790. doi:10.1002/jcb.27681. 30321449

[69] Xu X, Zhang M, Xu F, Jiang S. Wnt signaling in breast cancer: biological mechanisms, challenges and opportunities. Mol Cancer. 2020;19(1):165. doi:10.1186/s12943-020-01276-5. 33234169 PMC7686704

[70] Zhang Y, Wang X. Targeting the Wnt/β-catenin signaling pathway in cancer. J Hematol Oncol. 2020;13(1):165. doi:10.1186/s13045-020-00990-3. 33276800 PMC7716495

[71] Le PN, Keysar SB, Miller B, Eagles JR, Chimed T-S, Reisinger J, Gomez KE, Nieto C, Jackson BC, Somerset HL, et al. Wnt signaling dynamics in head and neck squamous cell cancer tumor-stroma interactions. Mol Carcinog. 2019; 58(3):398–410. doi:10.1002/mc.22937. 30378175 PMC6460915

[72] Mosa MH, Michels BE, Menche C, Nicolas AM, Darvishi T, Greten FR, Farin HF. A Wnt-induced phenotypic switch in cancer-associated fibroblasts inhibits EMT in colorectal cancer. Cancer Res. 2020;80(24):5569–5582. doi:10.1158/0008-5472.CAN-20-0263. 33055221

[73] Salaritabar A, Berindan-Neagoe I, Darvish B, Hadjiakhoondi F, Manayi A, Devi KP, Barreca D, Orhan IE, Süntar I, Farooqi AA, et al. Targeting hedgehog signaling pathway: paving the road for cancer therapy. Pharmacol Res. 2019; 141:466–480. doi:10.1016/j.phrs.2019.01.014. 30639373

[74] Xin M, Ji X, De La Cruz LK, Thareja S, Wang B. Strategies to target the hedgehog signaling pathway for cancer therapy. Med Res Rev. 2018;38(3):870–913. doi:10.1002/med.21482. 29315702

[75] Ma Z, Gao X, Shuai Y, Xing X, Ji J. The m6A epitranscriptome opens a new charter in immune system logic. Epigenetics. 2021;16(8):819–837. doi:10.1080/15592294.2020.1827722. 33070685 PMC8331015

[76] Han D, Liu J, Chen C, Dong L, Liu Y, Chang R, Huang X, Liu Y, Wang J, Dougherty U, et al. Anti-tumour immunity controlled through mRNA m(6)A methylation and YTHDF1 in dendritic cells. Nature. 2019; 566(7743):270–274. doi:10.1038/s41586-019-0916-x. 30728504 PMC6522227

[77] Gao Y, Vasic R, Song Y, Teng R, Liu C, Gbyli R, Biancon G, Nelakanti R, Lobben K, Kudo E, et al. m(6)A modification prevents formation of endogenous double-stranded RNAs and deleterious innate immune responses during hematopoietic development. Immunity. 2020; 52(6):1007–1021.e1008. doi:10.1016/j.immuni.2020.05.003. 32497523 PMC7408742

[78] Wang Q, Chen C, Ding Q, Zhao Y, Wang Z, Chen J, Jiang Z, Zhang Y, Xu G, Zhang J, et al. METTL3-mediated m 6 a modification of HDGF mRNA promotes gastric cancer progression and has prognostic significance. Gut. 2020; 69(7):1193–1205. doi:10.1136/gutjnl-2019-319639. 31582403

[79] Netea MG, Joosten LAB, Latz E, Mills KHG, Natoli G, Stunnenberg HG, O’Neill LAJ, Xavier RJ. Trained immunity: a program of innate immune memory in health and disease. Science. 2016;352(6284):aaf1098. doi:10.1126/science.aaf1098. 27102489 PMC5087274

[80] Wu SY, Fu T, Jiang Y-Z, Shao Z-M. Natural killer cells in cancer biology and therapy. Mol Cancer. 2020;19(1):120. doi:10.1186/s12943-020-01238-x. 32762681 PMC7409673

[81] Xie M, He J, He C, Wei S. γ secretase inhibitor BMS-708163 reverses resistance to EGFR inhibitor via the PI3K/Akt pathway in lung cancer. J Cell Biochem. 2015;116(6):1019–1027. doi:10.1002/jcb.25056. 25561332

[82] Lu H, Li Z-Y, Ding M, Liang C, Weng X-Q, Sheng Y, Wu J, Cai X. Trametinib enhances ATRA-induced differentiation in AML cells. Leuk Lymphoma. 2021;62(14):3361–3372. doi:10.1080/10428194.2021.1961231. 34355652

[83] Ni X, Hu G, Cai X. The success and the challenge of all-trans retinoic acid in the treatment of cancer. Crit Rev Food Sci Nutr. 2019;59(sup1):S71–s80. doi:10.1080/10408398.2018.1509201. 30277803

[84] Liu Y, Liu Q, Chen S, Liu Y, Huang Y, Chen P, Li X, Gao G, Xu K, Fan S, et al. APLNR is involved in ATRA-induced growth inhibition of nasopharyngeal carcinoma and may suppress EMT through PI3K-Akt-mTOR signaling. FASEB J. 2019; 33(11):11959–11972. doi:10.1096/fj.201802416RR. 31408612

[85] Porta C, Tortora G, Larkin JM, Hutson TE. Management of poor-risk metastatic renal cell carcinoma: current approaches, the role of temsirolimus and future directions. Future Oncol. 2016;12(4):533–549. doi:10.2217/fon.15.313. 26606910

[86] Widemann BC, Adamson PC. Understanding and managing methotrexate nephrotoxicity. Oncologist. 2006;11(6):694–703. doi:10.1634/theoncologist.11-6-694. 16794248

[87] Ji X, Guo H, Tang Q, Ma D, Xue W. A targeted nanocarrier based on polyspermine for the effective delivery of methotrexate in nasopharyngeal carcinoma. Mater Sci Eng C Mater Biol Appl. 2017. 81:48–56. doi:10.1016/j.msec.2017.07.036. 28888001

