# Supplementary figures and images for: M6A-mediated molecular patterns and tumor microenvironment infiltration characterization in nasopharyngeal carcinoma
Source: Cancer Biol Ther. 2024 Mar 26;25(1):2333590. doi: 10.1080/15384047.2024.2333590 (PMC10978033; doi:10.1080/15384047.2024.2333590)

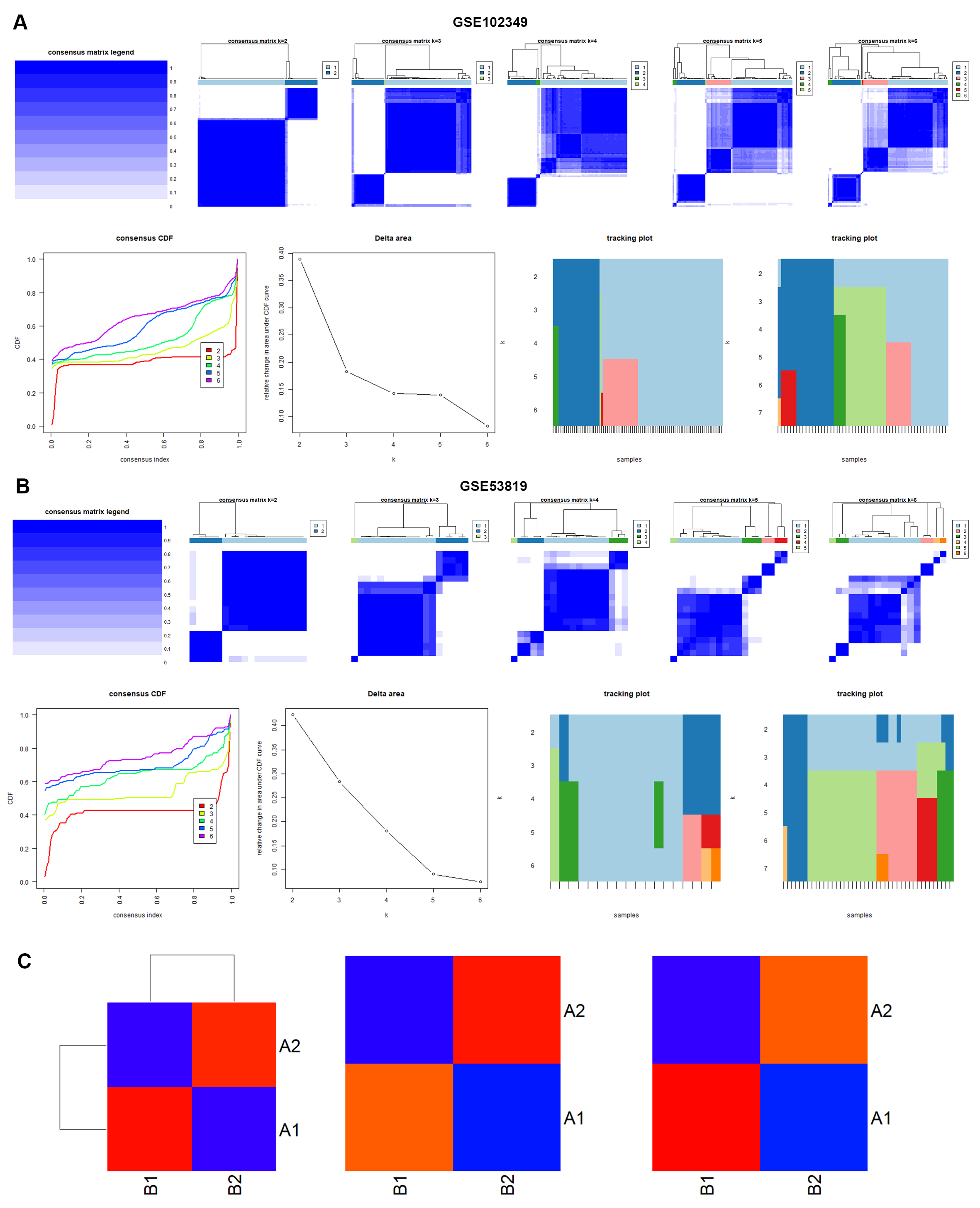

Supplement: Supplemental Material [file KCBT_A_2333590_SM2632.zip › Figure S1.tif]

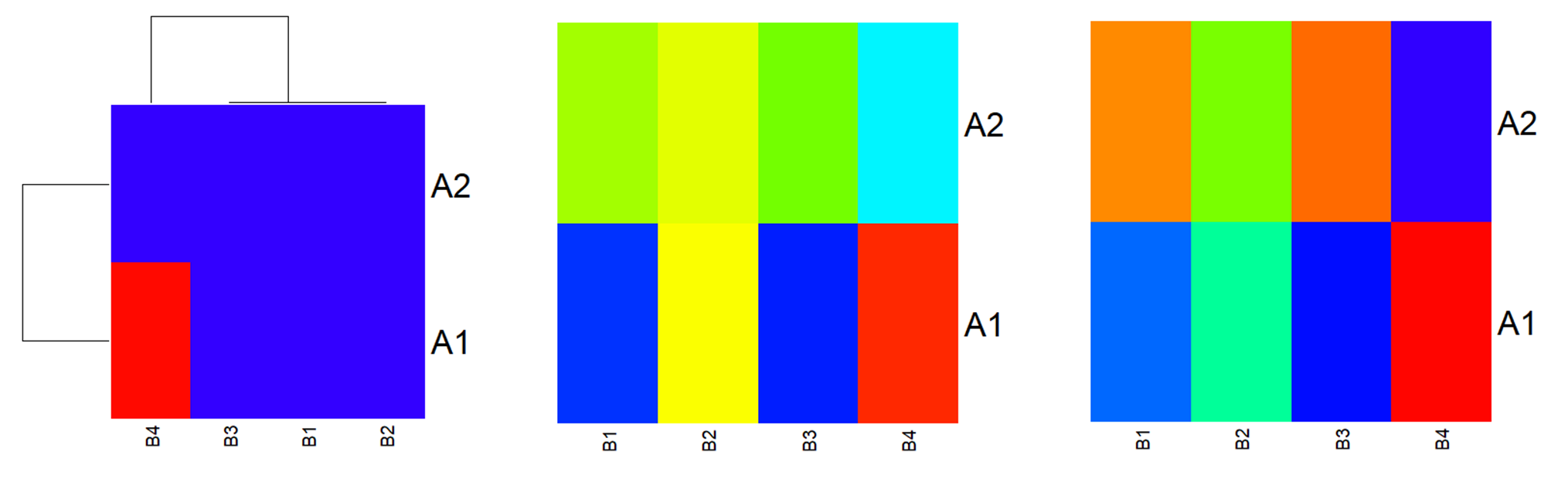

Supplement: Supplemental Material [file KCBT_A_2333590_SM2632.zip › Figure S2.tif]

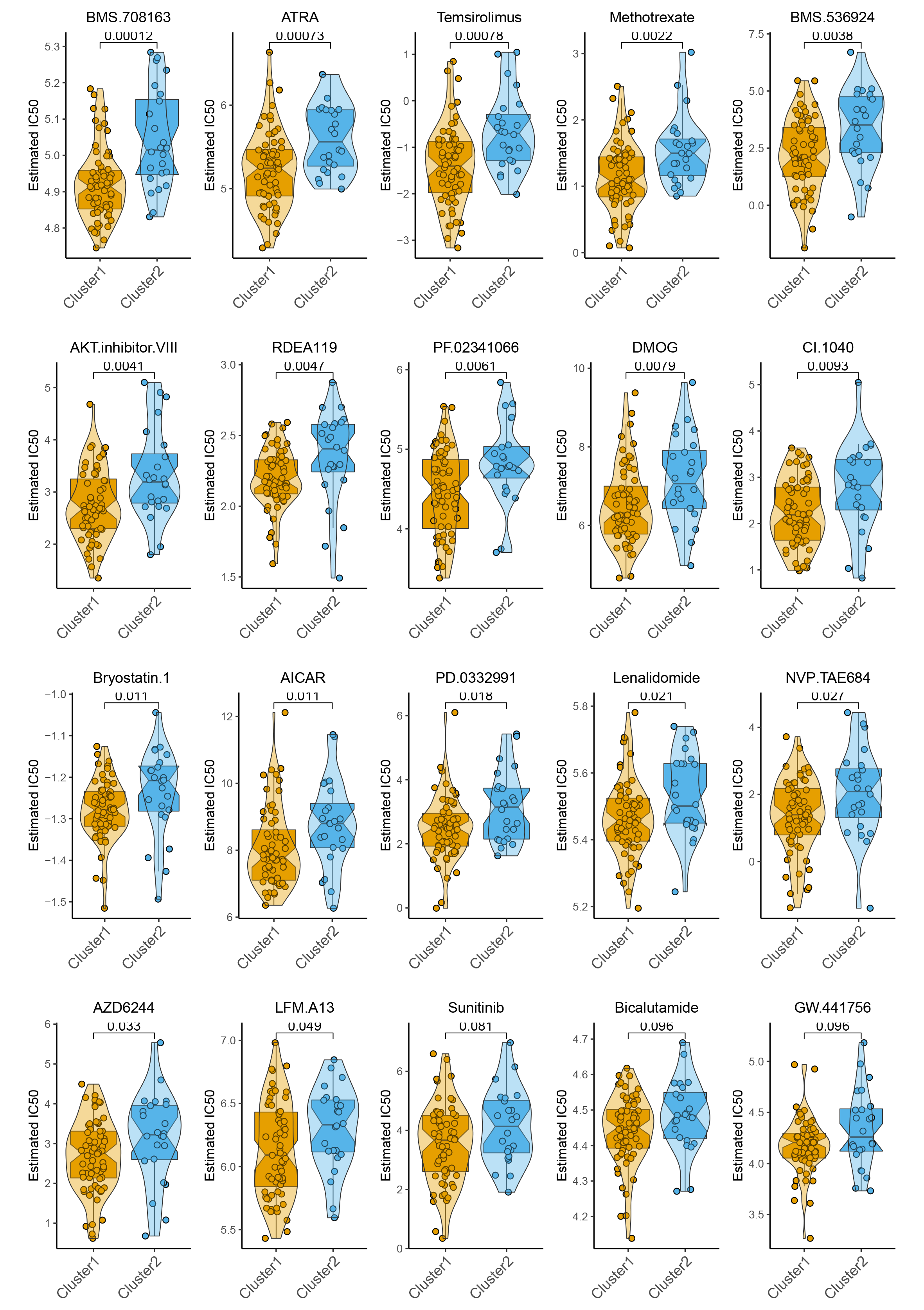

Supplement: Supplemental Material [file KCBT_A_2333590_SM2632.zip › Figure S4.tif]
